# Quality of life outcomes for people with serious mental illness living in supported accommodation: systematic review and meta-analysis

**DOI:** 10.1007/s00127-020-01885-x

**Published:** 2020-05-24

**Authors:** Michele Harrison, Anusua Singh Roy, Jenny Hultqvist, Ay-Woan Pan, Deborah McCartney, Nicola McGuire, Linda Irvine Fitzpatrick, Kirsty Forsyth

**Affiliations:** 1 School of Health Sciences, Queen Margaret University, Queen Margaret University Drive, Edinburgh, EH21 6UU UK; 2 Mental Health, Activity and Participation (MAP), Department of Health Sciences, Lund University, Lund, Sweden; 3 School of Occupational Therapy, College of Medicine, National Taiwan University, Taipei, Taiwan; 4 Present Address: Adult Learning Disability Service, Lynebank Hospital, Dunfermline, UK; 5 Present Address: Institute of Health and Wellbeing, University of Glasgow, Glasgow, UK; 6 Mental Health and Wellbeing, City of Edinburgh Health and Social Care Partnership, Edinburgh, UK

**Keywords:** Supported accommodation, Quality of life, Serious mental illness, Living conditions, Social functioning

## Abstract

**Purpose:**

To conduct a systematic review and meta-analysis of quality of life (QoL) outcomes for people with serious mental illness living in three types of supported accommodation.

**Methods:**

Studies were identified that described QoL outcomes for people with serious mental illness living in supported accommodation in six electronic databases. We applied a random-effects model to derive the meta-analytic results.

**Results:**

13 studies from 7 countries were included, with 3276 participants receiving high support (457), supported housing (1576) and floating outreach (1243). QoL outcomes related to wellbeing, living conditions and social functioning were compared between different supported accommodation types. Living condition outcomes were better for people living in supported housing *(*$g$= − 0.31; CI = [− 0.47; − 0.16]) and floating outreach ($g$= − 0.95; CI = [− 1.30; − 0.61]) compared to high-support accommodation, with a medium effect size for living condition outcomes between supported housing and floating outreach ($g$= − 0.40; CI = [− 0.82; 0.03]), indicating that living conditions are better for people living in floating outreach. Social functioning outcomes were significant for people living in supported housing compared to high support ($g$ = − 0.37; CI = [− 0.65; − 0.09]), with wellbeing outcomes not significant between the three types of supported accommodation.

**Conclusion:**

There is evidence that satisfaction with living conditions differs across supported accommodation types. The results suggest there is a need to focus on improving social functioning and wellbeing outcomes for people with serious mental illness across supported accommodation types.

**Electronic supplementary material:**

The online version of this article (10.1007/s00127-020-01885-x) contains supplementary material, which is available to authorized users.

## Introduction

Supported accommodation provides a place to live for people whose serious mental illness impacts on their self-care, social, occupational and cognitive functioning [1, 2]. It was developed in response to meeting the needs of this service user group following deinstitutionalisation [3]. Supported accommodation provides opportunities for people with serious mental illness to maintain a tenancy with varying levels of staff support provided to manage risk, develop and maintain living skills and engage in social and work activities [4]. Therefore, supported accommodation can have a range of functions providing a safe place to live, enabling people with serious mental illness to re-establish a sense of identity, have increasing choice and participate in activities and roles that give life meaning [5, 6]. The delivery of supported accommodation which enables individual’s recovery is influenced by a combination of service, contextual and relational factors [7]. There has been an increased focus on the effectiveness of supported accommodation [8] for people with serious mental illness and how it improves their quality of life (QoL). While contextual and service factors directly inform how supported accommodation is provided internationally, there are common features that are seen across all types of supported accommodation which relate to living arrangement (group or individual), level of staffing provided and type of support received [3, 4, 9].

It is expected that living in supported accommodation that provides increasing choice and control for people with serious mental illness will be reflected in improved quality of life (QoL) outcomes [10]. However, there is variable evidence regarding if people with serious mental illness do experience improved QoL outcomes between different types of supported accommodation provision. QoL can be considered to consist of a person’s general happiness with their life and the sense they have the life they want [11]. External life conditions can influence an individual’s satisfaction with their current life situation [12], particularly satisfaction with living conditions (living situation, income and finances, being in employment or education and general safety), and social functioning (relationships with family and others, health (physical and mental), leisure and social activities) [13]. Previous systematic reviews have considered QoL outcomes in supported accommodation as part of a range of psychosocial and health outcomes [14, 15], showing mixed results with regard to if supported accommodation improves QoL outcomes. However, QoL outcomes can be considered as providing a useful way of understanding if supported accommodation is meeting people’s expectations and supporting their recovery and there is a need to synthesise these findings to understand what type of supported accommodation optimises QoL outcomes for this group. Therefore, the aim of the systematic review and meta-analysis was to investigate if adults with serious mental illness experienced different QoL outcomes in three types of supported accommodation: (1) high support (2) supported housing and (3) floating outreach.

## Methods

We searched for studies that described Quality of life outcomes for people with serious mental illness living in all types of supported accommodation in six electronic databases: ProQUEST & ASSIA, the Cochrane Library, CINAHL, MEDLINE, PsycINFO and SCOPUS. For the database search, we used combinations of key words related to accommodation type and adults with severe mental illness informed by previous published systematic reviews [16, 17] ((resident* or hous* or accommod* or commun* or commu* or home*) AND (support* or support* or shelter*or outreach*) OR (residential treatm* or residential facility*) OR (supported hous* or public hous*) AND (Adult*) AND (Severe Mental Illness OR Persistent Mental illness; online resource: ESM_1). To ensure that we were able to locate all relevant research, search terms used were broad due to the variability in how supported accommodation is defined internationally. No restrictions were placed on publication dates to ensure all possible studies were reviewed and considered. The search was first completed in September 2016 and date of the last search was July 2019. Search terms were not updated. For reporting of the systematic review and meta-analysis, PRISMA guidelines were followed. For inclusion in the systematic review, studies had to meet the following criteria: (a) primary study; (b) studies that reported on interventions related to supported accommodation for service users with serious mental illness and (c) reported subjective and objective quality of life outcomes (subjective: wellbeing and life satisfaction; objective: living conditions and social functioning). Studies were excluded if they met the following criteria: (a) a validation study for a tool and (b) evaluation of an intervention (Online resource: ESM_2). We excluded tool validation studies as these focused on measurement of features of the living environment and intervention evaluations as these focused on adjunct interventions delivered to the participant population. In the case of duplicate studies, we selected the publication with the most information. For each study, the following data were extracted: sample size, diagnosis, mean age, ethnicity, country of recruitment, study design, and housing type using a form devised by the team (see Table 1). Initial data extraction was completed by M.H. and D.M.; A.S.R. completed extraction of means and standard deviations of QOL outcome scores (wellbeing, living conditions and social functioning).

**Table 1:** Data extraction table for studies included in systematic review and meta-analysis

Author, year, country	Sample size	Mean age	Diagnoses	Ethnicity	Supported accommodation type	QoL outcome			Study quality: measure/ rating
						Wellbeing	Living conditions	Social functioning	
Aubry et al. [35], Canada	930	39.4	Substance-related problems; psychotic disorder; major depressive disorder;	White, Aboriginal, Black, Asian, Other	Supported housing; floating outreach	Global wellbeing	Living situation, finances, safety	Leisure, social relations, family relations,	EPHPP Moderate
Brolin et al. [29], Sweden	370	49	Non-affective psychosis; affective psychosis; neuropsychiatric disabilities; other	Not reported	High support; supported housing	–	Security and privacy, control and choice about housing support	–	RTI Medium risk of bias
Brunt and Hansson [30] Sweden	51	High support: 39 Supported housing: 41	Psychosis	Not reported	High support; supported housing	–	Autonomy; practical orientation	Involvement, support, spontaneity	RTI Medium risk of bias
Brunt and Hansson [31] Sweden	76	High support: 39 Supported housing: 41	Psychosis	Not reported	High support; supported housing	Global wellbeing	Finances, living situation	Work; leisure activities, family relations, social relations, health	RTI Medium to low risk of bias
Chan et al. [36] Hong Kong	204	High support: 49.8 Supported housing: 50, floating outreach: 47.6	Inclusion criteria: people with diagnosis of schizophrenia (DSM-IV)	Chinese	High support; supported housing; floating outreach	Life satisfaction	Total life events	Physical (health), psychological, social relationship	RTI Medium to low risk of bias
De Heer Wunderink et al. [34] Netherlands	534	Supported housing: 43.1 floating outreach: 43.8	Schizophrenia; mood/anxiety disorders; substance abuse; personality disorder	Not reported	Supported housing; floating outreach	Global wellbeing	–	–	RTI Medium risk of bias
Jaeger et al. [33] Switzerland	168	High support: 45.1 Supported Housing: 45.1	Schizophrenia	Swiss, Schengen area, Non-Schengen area	High support; Supported housing	–	Activities of daily living, living conditions	Relationships, occupation/leisure	RTI Medium to low risk of bias

Author, year, country	Number of participants	Mean age	Diagnoses reported	Ethnicity	Supported accommodation type	QoL outcome			Study quality measure/rating
						Wellbeing	Living conditions	Social functioning	
Killaspy et al. [4] UK	619	46.1	Schizophrenia, schizo-affective disorder, bipolar affective disorder depression or anxiety; other	White	High support; Supported housing; Floating outreach	Global wellbeing	–	–	RTI low risk of bias
Lambri et al. [26] UK	80	41.6	Schizophrenia, bipolar disorder or other psychosis	African Caribbean, White British, European, South Asian	High support; Supported housing; Floating outreach	General wellbeing	Finances, living situation	Leisure, family relations, social relations, health, education	RTI Medium to low risk of bias
Muijen et al. [25] UK	189	High Support: 35 Supported Housing: 33	Schizophrenia; Mania; Depression; Neurosis;	British or Irish; Afro-Caribbean; Other	High support Supported housing	–	–	Social Functioning skills	EPHPP Strong
Mulholland et al. [28] UK	90	High support: 42.4, Supported Housing: 51.6 Floating Outreach: 40.4	Schizophrenia; personality disorder; major affective disorder; schizoaffective disorder; other	Not reported	High support; Supported housing; Floating outreach	–	Self-care skills, domestic skills	Social skills, community living skills	RTI Medium risk of bias
Simpson et al. [27] UK	34	High support: 45 Supported housing: 42, Floating outreach: 40	Schizophrenic psychosis, affective psychosis, other psychosis	Not reported	High support; Supported housing; Floating outreach	General wellbeing	Living situation, finances	Family relations, social relations, leisure, health	RTI Medium risk of bias
Yanos et al. [32] USA	44	47.2	Psychosis, bipolar disorder, major depression, other	White (not Hispanic), Black (not Hispanic), Hispanic; mixed, other, unknown	Supported housing; Floating outreach	–	Independence	Recreation, pro-social and occupational	RTI Medium risk of bias

The following three definitions of supported accommodation type [3, 4] were used to match supported accommodation described in included articles: (1) high-support accommodation is provided in hospital or the community with 24-h staffing on site. Meals, other daily living activities and supervision of medication are provided for people with serious mental illness; (2) supported housing is provided as tenancies in shared living with staff based on site up to 24 h a day. The focus is on rehabilitation, with people with serious mental illness being supported to gain independent living skills; (3) floating outreach services provide support to people with serious mental illness living in their own self-contained tenancy who are visited several times a week by support workers. Level of support will reduce as the individual becomes more able to look after themselves and their home. Matching was made by M.H. and A.S.R., based on living arrangement, number of hours staffing per week and type of input received from staff by residents.

QoL outcomes were matched to three QoL domains: wellbeing which included outcomes reported on overall happiness or satisfaction with current life situation or general wellbeing [18]; satisfaction with living conditions which included outcomes reported on satisfaction with living situation, employment, education, income/finances, general safety [13]; and satisfaction with social functioning which included outcomes reported on satisfaction with relationships with family and others, health (physical and mental), leisure and social activities [13].

The assessment of quality of papers and risk of bias was carried out by M.H., N.M., and D.M., using the Effective Public Health Practice Placement [EPHPP] Quality Assessment Tool for Quantitative Studies [19] for the two RCTs included in the meta-analysis. This provides an overall global rating of the quality of the study. 19 items of the Research Triangle Institute (RTI) item bank tool [20] were selected as relevant for establishing key areas of bias for observational studies, including information bias, selection bias and confounding [21, 22]. An indicative score to represent overall risk of bias was assigned following discussion between the reviewers (see Table 1).

A separate meta-analysis was conducted for each quality of life outcome comparing each pair of housing intervention, i.e. wellbeing, living conditions and social functioning for high support vs. supported housing, supported housing vs. floating outreach, and high support vs. floating outreach. This was done for all studies as well as for studies stratified by risk of bias. Effect size Hedges’ $g$ and corresponding variance ${V}_{g}$ are calculated for each study as follows:

$$g=\left(1- \frac{3}{4df-1}\right)\left(\frac{\stackrel{-}{{X}_{1}}- \overline{{X}_{2}}}{\sqrt{\frac{\left({n}_{1}-1\right){S}_{1}^{2}+\left({n}_{2}-1\right){S}_{2}^{2} }{{n}_{1}+ {n}_{2}-2}}}\right),$$

$$V_{g} = \left( {1 - ~\frac{3}{{4df - 1}}} \right)^{2} \left( {\frac{{n_{1} + n_{2} }}{{n_{1} n_{2} }} + \frac{{\left( {\frac{{\overline{{X_{1} }} - ~\overline{{X_{2} }} }}{{\sqrt {\frac{{\left( {n_{1} - 1} \right)S_{1}^{2} + \left( {n_{2} - 1} \right)S_{2}^{2} ~}}{{n_{1} + ~n_{2} - 2}}} }}} \right)^{2} }}{{2\left( {n_{1} + n_{2} } \right)}}} \right)$$

where $g$ is the unbiased estimate (especially for small sample sizes) of the standardized mean difference in outcome between two independent groups allocated to different housing types; ${V}_{g}$ is the uncertainty in the estimate of mean difference and within-group(s) standard deviation; $\stackrel{-}{{X}_{1}}$ and $\stackrel{-}{{X}_{2}}$ are the sample means of the two comparison groups; ${n}_{1}$ and ${n}_{2}$ are its respective sample sizes; *S*_1_ and *S*_2_ are the sample standard deviations of the two groups; and $df={n}_{1}+{n}_{2}-2$ is the degrees of freedom used in the estimation of the within-group(s) standard deviation [23].

Random-effects models that take into account variation between studies are fitted to obtain pooled estimates for wellbeing, living conditions and social functioning for different pairs of housing interventions. Similar to Cohen’s *d*, effect size Hedges’ $g$ is interpreted as 0.20—small, 0.50—medium and 0.80—large [23]. However, effect sizes should be interpreted cautiously, considering factors like quality of studies, uncertainty of estimates, results from previous relevant research in the field and feasibility and clinical importance of the findings [24]. The direction of $g$ indicates which housing model results in better wellbeing, living and social functioning for people with serious mental illness. Confidence interval for $g$ is indicative of the precision of the estimate. Wider the confidence interval, larger is the standard error and thus lesser the accuracy of the estimated $g$. A confidence interval inclusive of zero implies that the resulting effect is not statistically significant. Statistical significance indicates generalizability of the results since it implies that the effect $g$ observed is not due to random chance but an actual difference between the two sets of observations.

Heterogeneity between studies is assessed by Higgins’ *I*^2^ statistic, which measures the proportion of observed variance that is due to real differences in effect $g$ rather than sampling error (random chance). Potential sources of heterogeneity cannot be detected quantitatively using either subgroup analysis or meta-regression due to lack of sufficient number of studies reporting on relevant characteristics. Sensitivity analyses are conducted using the leave-one-out method to identify outliers or influential studies. Publication bias is examined by funnel plots using the trim and fill method (Online resource_ESM:3) although the results cannot be considered robust as the method is underpowered due to limited number of studies and sample size. Random-effects model outputs are visually represented through forest plots.

## Results

We identified 13 relevant studies published between 1989 and 2019. There were a total of 3276 people with serious mental illness in the included studies; 457 receiving high support, 1576 receiving supported housing and 1243 receiving floating outreach. Five studies were from the UK (four from England [10, 25–27] and one from Northern Ireland [28]), three studies from Sweden [29–31] and one study each from the USA [32], Switzerland [33], Netherlands [34], Canada [35] and Hong Kong [36]. 11 studies reported diagnosis of participants, with the remaining 2 referring to participants having serious mental illness. All 13 studies included supported housing, 11 studies included high-support accommodation and 7 studies included floating outreach accommodation. Seven studies reported wellbeing outcomes, ten studies reported living conditions outcomes and social functioning outcomes were reported in ten studies. A total of nine meta-analyses were conducted, one for each quality of life outcome and pair of supported accommodation types.

### High support vs. supported housing

**Fig. 1:**
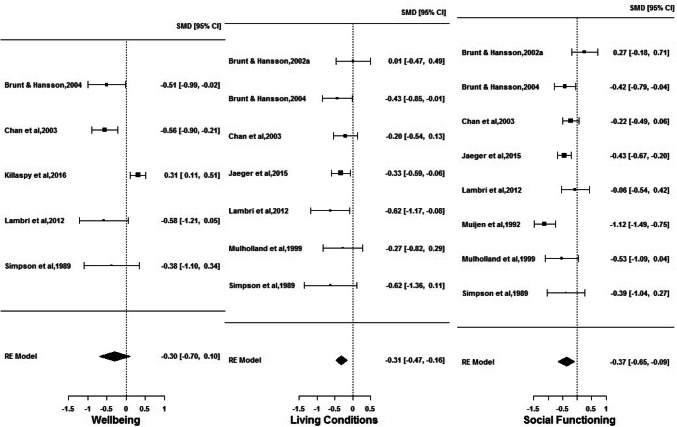
Comparison of wellbeing, living condition and social functioning outcomes for individuals in high support and supported housing

There were nine publications reporting on QOL outcomes in high support (*n* = 457) and supported housing (*n* = 1576). Five reported on wellbeing [10, 26, 27, 31, 36], seven on living conditions [26–28, 30, 31, 33, 36] and eight on social functioning [25–28, 30, 31, 33, 36]. Figure 1 is a set of forest plots depicting random-effects model results for all outcomes. A statistically significant $g$ was found for living conditions ($g$= − 0.31; CI = [− 0.47; − 0.16]) and social functioning ($g$= − 0.37; CI = [− 0.65; − 0.09]) which suggests that people living in supported housing have better living conditions and social functioning than those living in high-support settings. There is no evidence for a statistically significant difference in wellbeing between the two housing types ($g$= − 0.30; CI = [− 0.70; 0.10]). The size of all effects is small as per Cohen’s guidelines. Statistically significant heterogeneity between studies is found for outcomes wellbeing (*I*^2^ = 78.12%) and social functioning (*I*^2^ = 76.59%). Sensitivity analyses reveal influential studies to be Killaspy et al. [10] for wellbeing and Muijen et al. [25] for social functioning. Omission of Muijen et al. [25] produced no considerable change in inference, but omission of Killaspy et al. [10] resulted in a statistically significant $g$ for wellbeing ($g$= − 0.53; CI = [− 0.77; − 0.29]), thereby implying that people living in supported housing experience better wellbeing that those in high-support accommodation, with the size of this effect being medium. In terms of bias, for the wellbeing outcome, medium–low-risk studies [26, 31, 36] produced a significant medium effect, while the low-risk study Killaspy et al. [10] showed a significant small effect but in the opposite direction. For both living conditions and social functioning outcomes, medium–low-risk studies [26, 31, 33, 36] showed a significant small effect, whereas medium-risk studies [27, 28, 30] showed a non-significant small effect [Online resource ESM_4].

Publication bias in studies was found for QOL outcomes wellbeing and living conditions although it did not change the conclusions considerably.

The QOL outcome living conditions were further split into sub-categories finances and living situation to see if it affected the conclusion in any way (Online resource_ESM:7). Although living conditions overall results in a statistically significant difference of small effect size between people in high support and supported housing, its sub-category finances failed to exhibit a significant difference ($g$= − 0.31; CI = [− 0.66; 0.03]), while living situation showed a significant difference (*g* = − 0.50; CI = [− 0.96; − 0.05]) of medium effect size between the two models of housing with superior living situation being experienced by those in supported housing.

The QOL outcome social functioning was also further split into sub-categories social, leisure, family and health (Online resource_ESM:8). Although social functioning overall results in a statistically significant difference of small effect size between people living in high support and supported housing, its sub-category family failed to exhibit a significant difference ($g$= − 0.12; CI = [− 0.46; 0.23]), while social showed a significant difference (*g* = − 0.51; CI = [− 0.78; − 0.25]) of medium effect size, leisure showed a significant difference (*g* = -0.78; CI = [− 1.19; − 0.36]) of large effect size, and health showed a significant difference (*g* = − 0.22; CI = [− 0.39; − 0.06]) of small effect size between the two models of housing with superior outcomes experienced by those in supported housing.

### Supported housing vs. floating outreach services

**Fig. 2:**
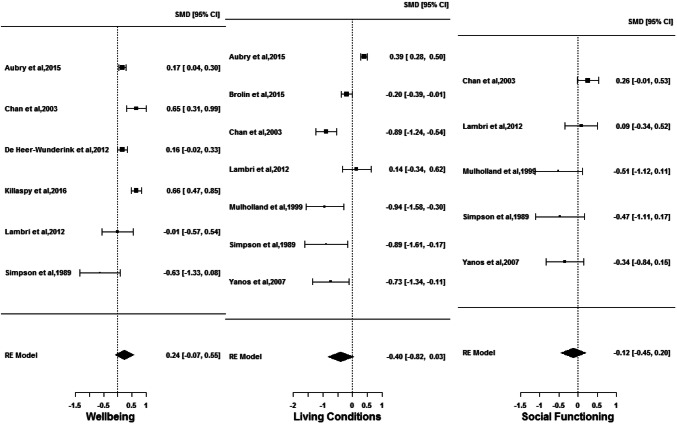
Comparison of wellbeing, living conditions and social functioning outcomes for individuals in supported housing and floating outreach

There were nine publications reporting on QOL outcomes in supported housing (*n* = 1576) and floating outreach (*n* = 1243). Six reported on wellbeing [10, 26, 27, 34–36], Seven on living conditions [26–29, 32, 35, 36] and five on social functioning [26–28, 32, 36]. Forest plots of the random-effects model results for the three QOL outcomes are in Fig. 2. Estimated $g$ for neither wellbeing ($g$ = 0.24; CI = [− 0.07; 0.55]), nor living conditions ($g$ = − 0.40; CI = [− 0.82; 0.03]) or social functioning ($g$ = − 0.12; CI = [− 0.45; 0.20]) achieved statistical significance, thereby indicating lack of evidence in rejecting the hypothesis that the overall quality of life experienced by people living in supported housing and floating outreach services are similar. Although not significant, the effects are small for wellbeing and social functioning, and close to medium for living conditions. In the presence of any difference, wellbeing seemed to be better for people living in supported housing, whereas living conditions and social functioning are better for people living in floating outreach. Statistically significant heterogeneity between studies is found for all the outcomes—wellbeing (*I*^2^ = 89.65%), living conditions (*I*^2^ = 92.41%) and social functioning (*I*^2^ = 59.57%). Sensitivity analyses reveal influential studies to be Aubry et al. [35] for living conditions and Chan et al. [36] for social functioning. Omission of Aubry et al. [35] results in a statistically significant $g$ for living conditions ($g$ = − 0.54; CI = [− 0.91; − 0.17]), thereby implying that people living in floating outreach experienced better living conditions than those in supported housing, with the size of this effect being medium. Omission of Chan et al. [36], however, produces no considerable change in the results. Publication bias in studies is found for QOL outcomes wellbeing and social functioning, although it does not change the conclusions considerably. No reasonable differences are observed on splitting QOL outcomes into sub-categories. In terms of bias, for the wellbeing outcome, Killaspy et al. [10] showed a medium significant effect, while both medium–low- [26, 36] and medium-risk studies [27, 34] showed non-significant effects, but in opposite directions. For the living conditions outcome, medium-risk studies [27–29, 32] showed a significant medium effect whereas medium–low-risk studies [26, 36] found a non-significant small effect, and for the social functioning outcome, medium-risk studies [27, 28, 32] showed a significant small effect while medium–low-risk studies [26, 36] showed a borderline significant small effect but in the opposite direction [Online resource ESM_5].

### High support vs. floating outreach services

**Fig. 3:**
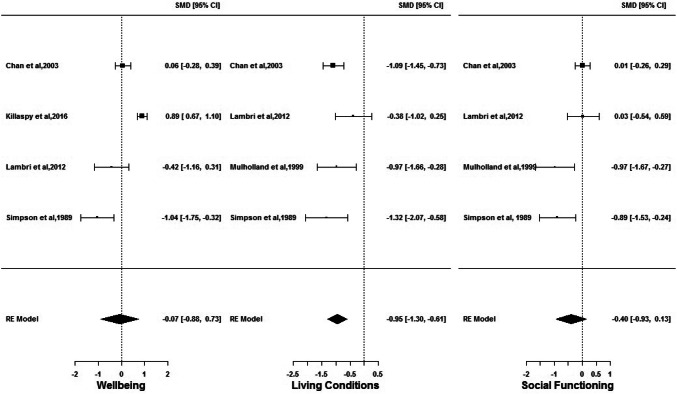
Comparison of wellbeing, living conditions and social functioning outcomes for individuals in high support and floating outreach

Five publications reported on QOL outcomes in high support (*n* = 457) and floating outreach (*n* = 1243). Four reported on wellbeing [10, 26, 27, 36], four on living conditions [26–28, 36] and four on social functioning [26–28, 36]. Figure 3 is a set of forest plots depicting the random-effects model results for the three outcomes. A statistically significant $g$ with large effect size is found for living conditions only ($g$= − 0.95; CI = [− 1.30; − 0.61]), indicating that people living in floating outreach services experience greatly enhanced living conditions than those in high-support settings. Non-significant and very small effect for wellbeing (*g* = − 0.07; CI = [− 0.88; 0.73]) and small effect for social functioning ($g$= − 0.40; CI = [− 0.93; 0.13]) imply lack of evidence in rejecting the hypothesis that wellbeing and social functioning experienced by people living in high support and floating outreach services are similar. In the presence of any difference, both QOL outcomes seem to be better for people living in floating outreach. Statistically significant heterogeneity between studies is found for wellbeing (*I*^2^ = 93.32%) and social functioning (*I*^2^ = 76.04%) only. Sensitivity analyses reveal influential studies to be Killaspy et al. [10] and Simpson et al. [27] for wellbeing, Lambri et al. [26] for living conditions and Chan et al. [36] for social functioning. Omission of neither study resulted in anything different from what had already been observed. Publication bias in studies is found only for social functioning, although it does not change the conclusions considerably. No reasonable differences are observed on splitting QOL outcomes into sub-categories. In terms of bias, for the wellbeing outcome, low- [10] and medium-risk studies [27] showed a large significant effect, but in opposite directions, whereas medium–low-risk studies [26, 36] showed a non-significant effect. For the living conditions outcome, there was no substantial difference in size, direction and significance of effect between medium-risk [27, 28] and medium–low-risk [26, 36] studies and for the social functioning outcome medium-risk studies [27, 28] showed a significant large effect whereas medium–low-risk studies [26, 36] produced a non-significant effect [Online resource ESM_6].

## Discussion

This review of 13 studies investigated the performance of three types of supported accommodation interventions on QOL outcomes: wellbeing, living conditions and social functioning for adults with serious mental illness. Statistically significant heterogeneity with high *I*^2^ statistics are found for the majority of the meta-analyses conducted. Sensitivity analyses reveal six out of thirteen studies to be outliers across all meta-analyses performed; however, the majority of these do not cause any change in inference upon omission (Online resource_ESM:9). We provide a discussion of the results considering the wider context of research on QoL outcomes for adults with serious mental illness living in supported accommodation.

### High-support accommodation

High-support accommodation is found to offer the least favourable quality of life to people in comparison to both supported housing and floating outreach. The meta-analysis showed that satisfaction with living condition outcomes were better for people living in supported housing and floating outreach compared to high-support accommodation, with subgroup analysis for supported housing showing a medium effect size for satisfaction with living situation. The potential reasons for this difference are that the purpose of high-support accommodation differs from supported housing and floating outreach. High-support accommodation is typically provided for people who are experiencing a high level of symptoms, which are having a significant impact on their ability to look after themselves and manage their daily lives. Twenty-four-hour care is provided to create a safe environment to manage risk with staff delivering routine daily living activities. There are mixed experiences of this type of support, with some people identifying that it is helpful in providing safety and stability [10]. Other people experience high-support accommodation as restrictive and reducing autonomy [37]. The contrast between this type of living environment and the two other types of supported accommodation included in the meta-analyses is significant in that people living in supported housing and floating outreach have increased choices about how they manage their living environment and organise their daily routine. This has been shown to positively impact on satisfaction with living conditions [38–43] with perception of the physical environment and having a positive social climate also being influential [44].

Social functioning outcomes were also better in supported housing than high-support accommodation, particularly in social and leisure subcategories. The increased rehabilitative focus of supported housing focuses on people with serious mental illness increasing participation in social and leisure activities [4]. Satisfaction with activities is shown to be positively related to level of participation in activities [45, 46]. Lengthy stays in high-support accommodation can increase dependency on staff and services [47] and result in reduced opportunities to participate in social and leisure activities within established social networks outside the high-support environment [48].

Wellbeing outcomes were not significant between high support and the other types of supported accommodation. Killaspy et al.’s study was influential in the meta-analyses, with wellbeing outcomes showing a moderate effect size in high support compared with supported housing or floating outreach. This is in contrast to the other studies in the meta-analyses which showed that wellbeing outcomes had a moderate effect size in supported housing and floating outreach. We were unable to establish what informed this difference as other factors that are reported as impacting on wellbeing outcomes, including the impact of negative symptoms, e.g. motivation and depression [49–51] and having a greater number of unmet needs [52–54] were not reported in all of the included studies.

### Supported housing and floating outreach accommodation

There was an absence of a significant difference in all QoL outcomes between supported housing and floating outreach accommodation. By definition, floating outreach accommodation provides the greatest opportunity for people with serious mental illness to have choice and control of their life, which was confirmed in Killaspy et al.’s study [10]. However, they can be more socially isolated as a result of living alone and involved in less social activity [29, 55]. This can contribute to people with serious mental illness feeling less safe and secure in their homes [56] potentially impacting on satisfaction with living conditions. It has also been reported that initial gains in social functioning made by people with serious mental illness in supported housing are generally maintained but do not increase over time [57], potentially explaining the lack of significant difference between social functioning outcomes.

### Limitations

Our study has a number of limitations. The number of studies included in each meta-analysis is small, ranging between 3 and 8 [58, 59]. This can result in underestimation of the average population effect size and average sampling error [60]. With a limited number of studies, the confidence intervals from random-effects models are wider and statistical power lower leading to results that need to be interpreted with caution. Accurate analyses of between-study variance require meta-analyses based on a substantial number of studies which were not available to us.

Heterogeneity is a recognized feature of meta-analyses which needs careful consideration when including observational studies. Consideration of heterogeneity arising from study design and risk of bias are recommended [20]. Assessment of information bias, selection bias and confounding across the included observational studies using the RTI Item Bank ensured that these were considered for all included observational studies [19, 20]. The inclusion of studies with different experimental designs is also justified when appropriate quality assessment is completed [61]. However, it is acknowledged that even with accurate identification of bias, the introduction of heterogeneity is unavoidable in meta-analysis [62]. We recognise that the grouping of supported accommodation type based on published definitions of supported accommodation which incorporated living arrangement, level of staffing provided and type of support did not account for the different service focus (move on/not move on) as identified in McPherson’s taxonomy [9], which could affect how QoL outcomes were rated by study participants. This taxonomy was not available when the systematic review and meta-analysis was first conceptualised. Inconsistent reporting of demographic data across the included studies meant that other potential sources of heterogeneity could not be analysed [63]. Publication bias assessed from funnel plots using the trim and fill method produced results that are not adequately reliable due to not meeting the rule of thumb of at least ten studies [64] (Online resource_ESM:3).

## Conclusions

QoL outcomes can provide an indication of how satisfied people with serious mental illness are living in different types of supported accommodation. The meta-analysis showed that satisfaction with living conditions was significantly different between the three types of supported accommodation. The results of our study suggest that there is a need to focus on improving social functioning and wellbeing outcomes across supported accommodation types. There is also a need to further identify the factors which create positive living conditions for people which balance managing risk, developing daily living skills and enabling increased choice and autonomy for service users to create supported accommodation that enables recovery for people with serious mental illness.

## Electronic supplementary material

Below is the link to the electronic supplementary material.

Supplementary file1 (DOCX 2572 kb)

## References

[1] National Institute of Mental Health (1997). Towards a model for a comprehensive community-based mental health system.

[2] Cook S, Chambers E (2009). What helps and hinders people with psychotic conditions doing what they want in daily life?. Br J Occup Ther.

[3] Priebe S, Saidi M, Want A, Mangalore R, Knapp M (2009). Housing services for people with mental disorders in England: patient characteristics, care provision and costs. Soc Psychiatry Psychiatr Epidemiol.

[4] Killaspy H, White S, Dowling S, Krotofil J, McPherson P, Sandhu S (2016). Adaptation of the quality indicator for rehabilitative care (QuIRC) for use in supported accommodation services (QuIRC-SA). BMC Psychiatry.

[5] Browne G, Hemsley M, St JW (2008). Consumer perspectives on recovery: a focus on housing following discharge from hospital. Int J Ment Health Nurs.

[6] Piat M, Seida K, Padgett D (2019). Choice and personal recovery for people with serious mental illness living in supported housing. J Ment Health.

[7] Krotofil J, McPherson P, Killaspy H (2019). Service user experiences of specialist mental health supported accommodation: a systematic review of qualitative studies and narrative synthesis. Health Soc Care Community.

[8] Killaspy H, Priebe S, King M, Eldridge S, McCrone P, Shepherd G, et al (2019) Supported accommodation for people with mental health problems: the QuEST research programme with feasibility RCT. *Programme Grants Appl Res* **7**:7. London: NIHR 31553550

[9] McPherson P, Krotofil J, Killaspy H (2018). What works? Toward a new classification system for mental health supported accommodation services: the simple taxonomy for supported accommodation (STAX-SA). Int J Environ Res Public Health.

[10] Killaspy H, Priebe S, Bremner S, McCrone P, Dowling S, Harrison I (2016). Quality of life, autonomy, satisfaction, and costs associated with mental health supported accommodation services in England: a national survey. Lancet.

[11] Salvador-Carulla L, Lucas R, Ayuso-Mateos JL, Miret M (2014). Use of the terms “Wellbeing” and “Quality of Life” in health sciences: a conceptual framework. Eur J Psychiat.

[12] Hansson L (2006). Determinants of quality of life in people with severe mental illness. Acta Psychiatr Scand Suppl.

[13] Barry MM, Zissi A (1997). Quality of life as an outcome measure in evaluating mental health services: a review of the empirical evidence. Soc Psychiatry Psychiatr Epidemiol.

[14] Kyle T, Dunn JR (2008). Effects of housing circumstances on health, quality of life and healthcare use for people with severe mental illness: a review. Health Soc Care Community.

[15] McPherson P, Krotofil J, Killaspy H (2018). Mental health supported accommodation service: a systematic review of mental health and psychosocial outcomes. BMC Psychiatry.

[16] Leff SH, Chow CM, Pepin R, Conley J, Allen IE, Seaman CA (2009). Does one size fit all? What we can and cannot learn from a meta-analysis of housing models for persons with mental illness. Psychiatr Serv.

[17] Chilvers P, Macdonald G, Hayes A (2006) Supported housing for people with severe mental disorders. Cochrane Database of Syst Rev CD000453 10.1002/14651858.CD000453 12519544

[18] Fakhoury WKH, Priebe S (2002). Subjective quality of life; its association with other constructs. Int Rev Psychiatry.

[19] Thomas BH, Ciliska D, Dobbins M, Micucci S (2004). A process for systematically reviewing the literature: providing the research evidence for public health nursing interventions. Worldviews Evid Based Nurs.

[20] Viswanathan M, Berkman ND (2012). Development of the RTI item bank on risk of bias and precision of observational studies. J Clin Epidemiol.

[21] Dekkers OM, Vandenbroucke JP, Cevallos M, Renehan AG, Altman DG, Egger M (2019). COSMOS-E: guidance on conducting systematic reviews and meta-analyses of observational studies of etiology. PLoS Med.

[22] Mueller M, D’Addario M, Egger M, Cevallos M, Dekkers O, Mugglin C (2018). Methods to systematically review and meta-analyse observational studies: a systematic scoping review of recommendations. BMC Med Res Methodol.

[23] Borenstein M, Hedges LV, Higgins J, Rothstein HR (2009). Introduction to meta-analysis.

[24] Cohen J (1988). Statistical power analysis for the behavioral sciences.

[25] Muijen M, Marks IM, Connolly J, Audini B (1992). Home based care and standard hospital care for patients with severe mental illness: a randomised controlled trial. BMJ.

[26] Lambri M, Chakraborty A, Leavey G, King M (2012) Quality of life and unmet need in people with psychosis in the London Borough of Haringey, UK. Sci World J 836067 10.1100/2012/836067 PMC3506896 23213300

[27] Simpson CJ, Hyde CE, Faragher EB (1989). The chronically mentally ill in community facilities: a study of quality of life. Br J Psychiatry.

[28] Mulholland C, Wilson C, McCrum B, MacFlynn G (1999). Factors influencing the support offered to the seriously mentally ill by a community rehabilitation service. J Ment Health.

[29] Brolin R, Rask M, Syrén S, Baigi A, Brunt DA (2015). Satisfaction with housing and housing support for people with psychiatric disabilities. Issues Ment Health Nurs.

[30] Brunt D, Hansson L (2002). A comparison of the psychosocial environment of two types of residence for persons with severe mental illness: small congregate community residences and psychiatric inpatient settings. Int J Soc Psychiatry.

[31] Brunt D, Hansson L (2004). The quality of life of persons with severe mental illness across housing settings. Nord J Psychiatry.

[32] Yanos PT, Felton BJ, Tsemberis S, Frye VA (2007). Exploring the role of housing type, neighborhood characteristics, and lifestyle factors in the community integration of formerly homeless persons diagnosed with mental illness. J Ment Health.

[33] Jaeger M, Briner D, Kawohl W, Seifritz E, Baumgartner-Nietlisbach G (2015). Psychosocial functioning of individuals with schizophrenia in community housing facilities and the psychiatric hospital in Zurich. Psychiatry Res.

[34] De Heer-Wunderink C, Visser E, Caro-Nienhuis A, Sytema S, Wiersma D (2012). Supported housing and supported independent living in the Netherlands, with a comparison with England. Community Ment Health J.

[35] Aubry T, Tsemberis S, Adair CE, Veldhuizen S, Streiner D, Latimer E (2015). One-year outcomes of a randomized controlled trial of Housing First with ACT in five Canadian cities. Psychiatr Serv.

[36] Chan GWL, Ungvari GS, Shek DTL, Leung Dagger JJP (2003). Hospital and community-based care for patients with chronic schizophrenia in Hong Kong—quality of life and its correlates. Soc Psychiatry Psychiatr Epidemiol.

[37] Bredski J, Forsyth K, Harrison M, Mountain D, Irvine L, Maciver D (2015). What in-patients want: a qualitative study of what’s important to mental health service users in their recovery (Wayfinder Partnership). Ment Health Rev.

[38] Nelson G, Hall GB, Walsh-Bowers R (1998). The relationship between housing characteristics, emotional wellbeing and the personal empowerment of psychiatric consumer/survivors. Community Ment Health J.

[39] Hobbs C, Newton L, Tennant C, Rosen A, Tribe K (2002). Deinstitutionalization for long-term mental illness: a 6-year evaluation. Aust N Z J Psychiatry.

[40] Padgett DK (2007). There’s no place like (a) home: ontological security among persons with serious mental illness in the United States. Soc Sci Med.

[41] Piat M, Lesage A, Boyer R, Dorvil H, Couture A, Grenier G (2008). Housing for persons with serious mental illness: consumer and service provider preferences. Psychiatr Serv.

[42] Kloos B, Shah S (2009). A social ecological approach to investigating relationships between housing and adaptive functioning for persons with serious mental illness. Am J Community Psychol.

[43] Mannix-McNamara P, Eichholz EC, Vitale A, Hourdan D (2012). Experiences of clients who have made the transition from the psychiatric hospital to community service provision: a phenomenological approach. Int J Ment Health Promot.

[44] Marcheschi E, Laike T, Brunt D, Hansson L, Johansson M (2015). Quality of life and place attachment among people with severe mental illness. J Environ Psychol.

[45] Eklund M (2009). Work status, daily activities and quality of life among people with severe mental illness. Qual Life Res.

[46] Sánchez J, Frain MP, Bezyack JP, Rosenthal DA, Tansey TN (2016). Predicting quality of life in adults with severe mental illness: extending the international classification of functioning. Disabil Health Rehabil Psychol.

[47] Loch AA (2014). Discharged from a mental health admission ward: is it safe to go home? A review of the negative outcomes of psychiatric hospitalization. Psychol Res Behav Manag.

[48] Dickinson D, Green G, Hayes C, Gilheaney B, Whittaker A (2002). Social network and social support characteristics amongst individuals recently discharged from acute psychiatric units. J Psychiatr Ment Health Nurs.

[49] Ruggeri M, Gater R, Bisoffi G, Barbui C, Tansella M (2002). Determinants of subjective quality of life in patients attending community-based mental health services; the South-Verona Outcome Project 5. Acta Psychiatr Scand.

[50] Fleury M, Grenier G, Bamvita J (2018). Associated and mediating variables related to quality of life among service users with mental disorders. Qual Life Res.

[51] Saperia S, Da Silva S, Siddiqui I, McDonald K, Agrid O, Remington G (2018). Investigating the predictors of happiness, life satisfaction and success in schizophrenia. Compr Psychiatry.

[52] Hansson L, Björkman T (2007). Are factors associated with subjective quality of life in people with severe mental illness consistent over time?—A 6-year follow-up study. Qual Life Res.

[53] Ritsner MS, Arbitman M, Lisker A, Ponizovsky AM (2012). Ten year Quality of Life outcomes among patients with schizophrenia and schizoaffective disorders. II. Predictive value of psychosocial factors. Qual Life Res.

[54] Emmerink PMJ, Roeg DPK (2016). Predictors of quality of life of people receiving intensive community based care. Qual Life Res.

[55] Eklund M, Argentzell E, Bejerholm U, Tjörnstrand C, Brunt D (2017). Wellbeing, activity and housing satisfaction—comparing people with psychiatric disabilities in supported housing and ordinary housing with support. BMC Psychiatry.

[56] Whitley R, Harris M, Drake RE (2008). Safety and security in small scale recovery housing for people with severe mental illness: an inner city case study. Psychiatr Serv.

[57] McInerney SJ, Finnerty S, Avalos G, Walsh E (2010). Better off in the community? A 5-year follow up study of long-term psychiatric patients discharged into the community. Soc Psychiatry Psychiatr Epidemiol.

[58] Higgins JP, Thompson SG, Spiegelhalter DJ (2009). A re-evaluation of random-effects meta-analysis. J R Stat Soc Ser A (Statistics in Society).

[59] Bender R, Friede T, Koch A, Kuss O, Schlattmann P, Schwarzer G, Skipka G (2018). Methods for evidence synthesis in the case of very few studies. Res Synth Methods.

[60] Durlak JA (1999). How to select, calculate, and interpret effect sizes. J Pediatr Psychol.

[61] Shrier I, Boivin JF, Steele RJ, Platt RW, Furlan A, Kakuma R, Brophy J, Rossignoi M (2007). Should meta-analyses of interventions include observational studies in addition to randomized control trials? A critical examination of underlying principles. Am J Epidemiol.

[62] Ioannidis JP, Patsopoulos NA, Evangelou E (2007). Uncertainty in heterogeneity estimates in meta-analyses. BMJ.

[63] Higgins J, Thompson SG, Deeks JJ, Altman DG (2003). Measuring inconsistency in meta-analyses. BMJ.

[64] Sterne JA, Sutton AJ, Ionnidis JP, Terrin N, Jone DR, Lau J, Carpenter J, Rücker G, Harbord RM, Schmid CH, Tetzlaff J, Deeks JJ, Peters J, Macaskill P, Schwarzer G, Duval S, Altman DG, Moher D, Higgins JPT (2011). Recommendations for examining and interpreting funnel plot asymmetry in meta-analyses of randomised controlled trials. BMJ.

