# Supplementary material for: Quality of life outcomes for people with serious mental illness living in supported accommodation: systematic review and meta-analysis
Source: Soc Psychiatry Psychiatr Epidemiol. 2020 May 24;55(8):977–88. doi: 10.1007/s00127-020-01885-x (PMC7395041; doi:10.1007/s00127-020-01885-x)

Online resource.

ESM1: Search terms

ESM2: PRISMA Flow diagram

ESM3: Funnel Plots for Publication Bias

ESM4: Stratified Forest Plots: High Support vs Supported Housing

ESM5: Stratified Forest Plots: Supported Housing vs Floating Outreach

ESM6: Stratified Forest Plots: High Support vs Floating Outreach

ESM7: Comparison of Living Conditions outcomes for individuals in High Support and Supported

Housing

ESM8: Comparison of Social Functioning outcomes for individuals in High Support and Supported Housing

ESM9: Sensitivity analyses

ESM1: Example search for systematic review

**Search Strategy: PsycInfo**

1. resident*
2. hous*
3. accommod*
4. commun*
5. commu*
6. home*
7. 1 or 2 or 3 or 4 or 5 or 6
8. support*
9. shelter*
10. outreach*
11. 8 or 9 or 10
12. 7 and 11
13. residential treatm*
14. residential facility*
15. 13 or 14
16. supported hous*
17. public hous*
18. 16 or 17
19. 12 or 15 or 18
20. adult*
21. severe mental illness
22. persistent mental illness
23. 21 or 22
24. 19 and 20 and 23


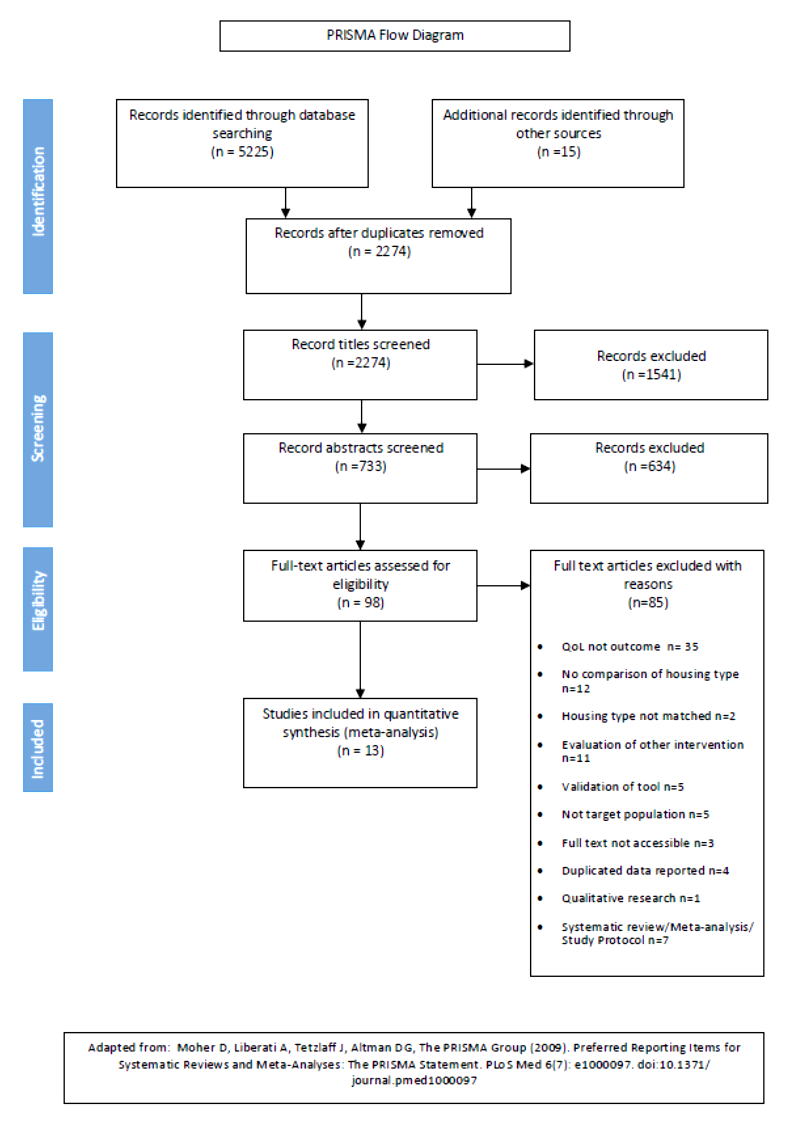


ESM2: PRISMA Flow diagram

ESM3: Funnel Plots for Publication Bias

ESM3.1 High Support vs Supported Housing – Wellbeing


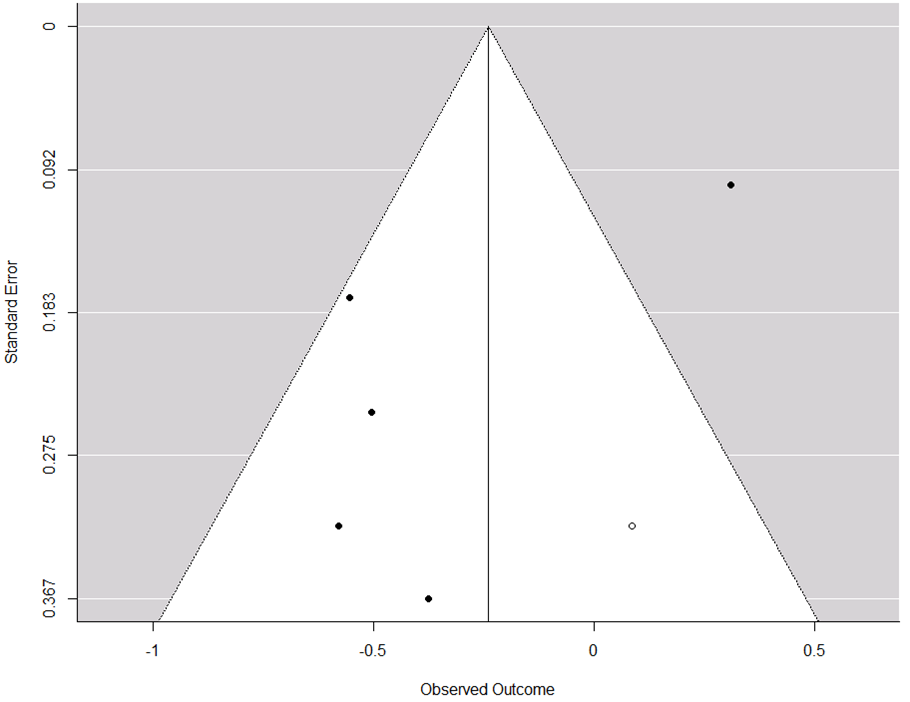


ESM3.2 High Support vs Supported Housing – Living Conditions


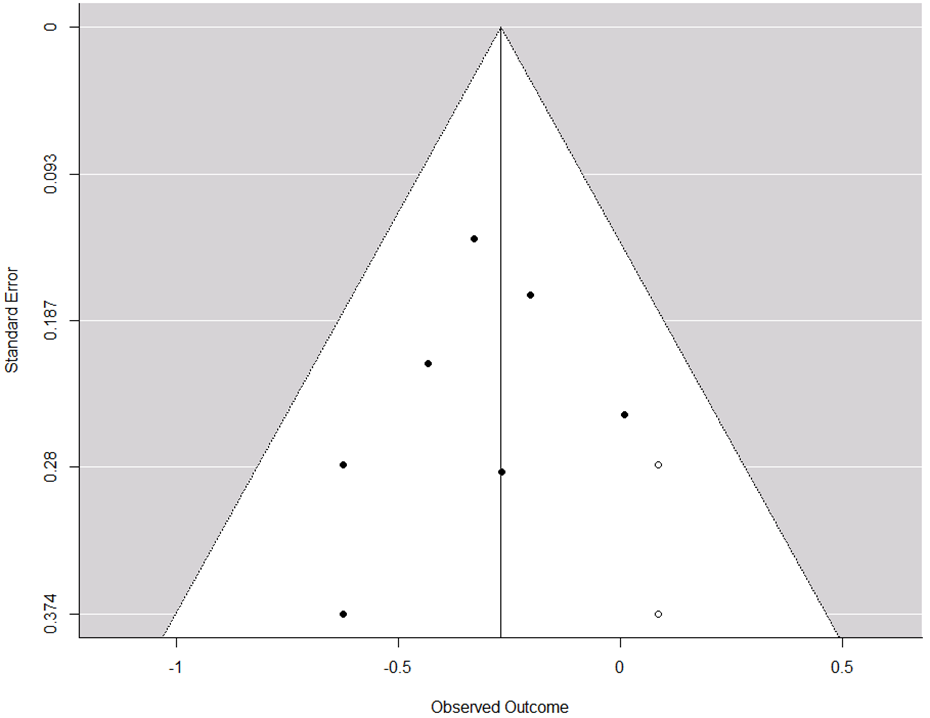


ESM3.3 High Support vs Supported Housing – Social Functioning


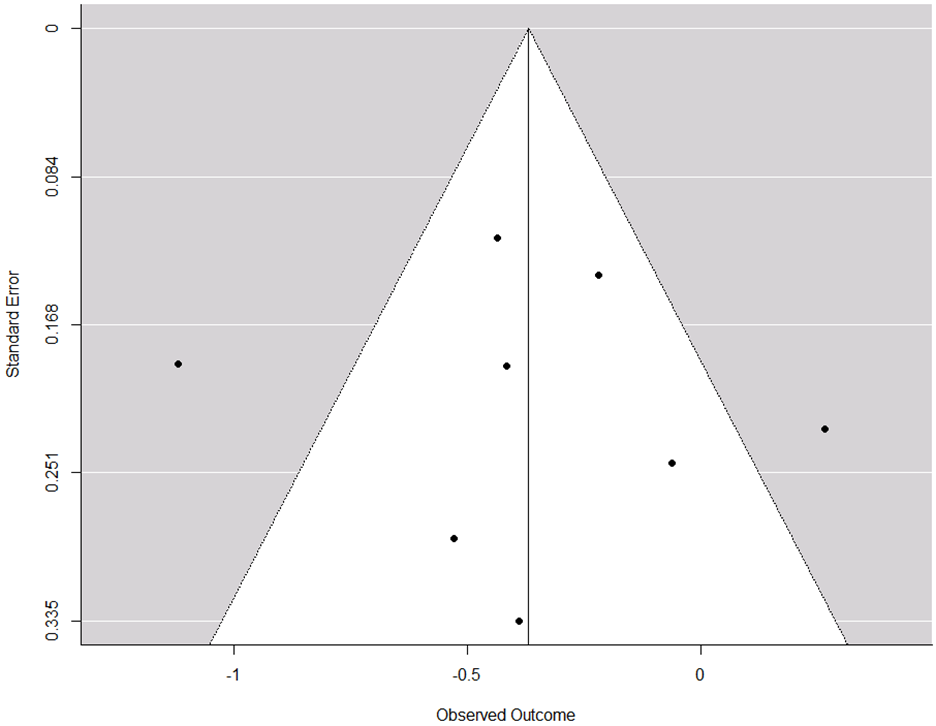


ESM3.4 Supported Housing vs Floating Outreach Services – Wellbeing


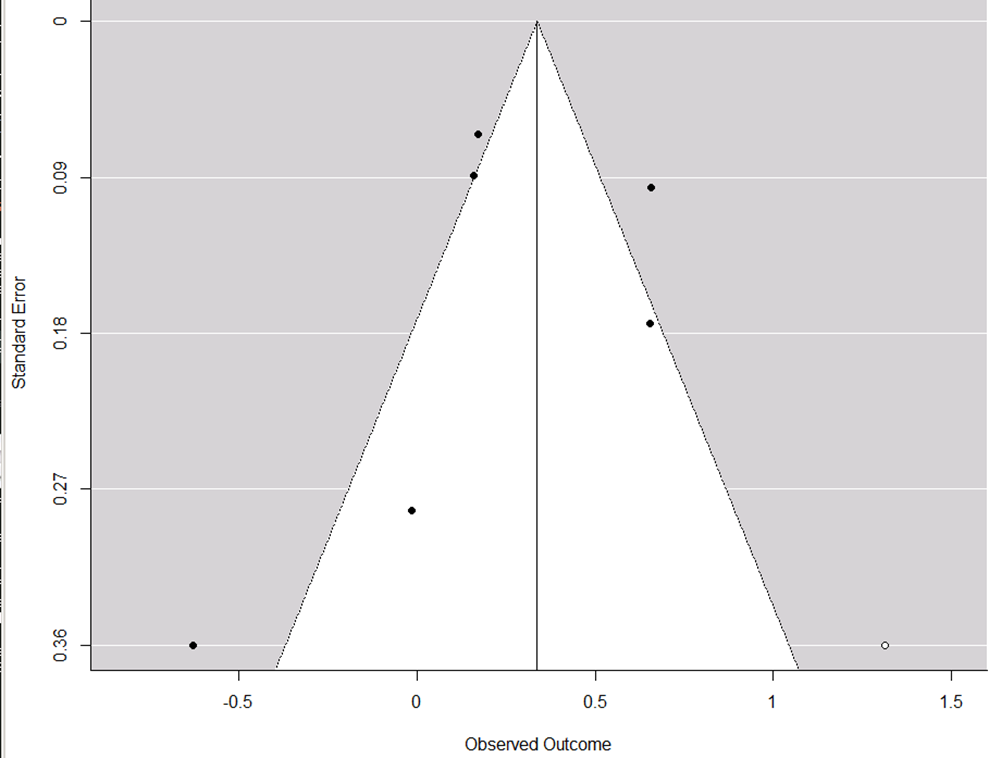


ESM3.5 Supported Housing vs Floating Outreach Services – Living Conditions


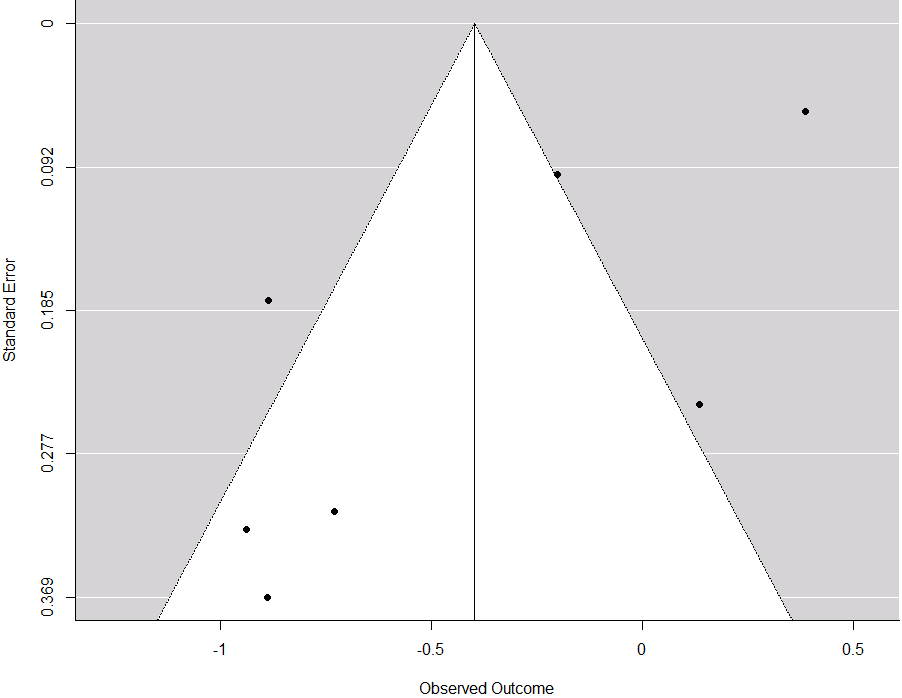


ESM3.6 Supported Housing vs Floating Outreach Services – Social Functioning


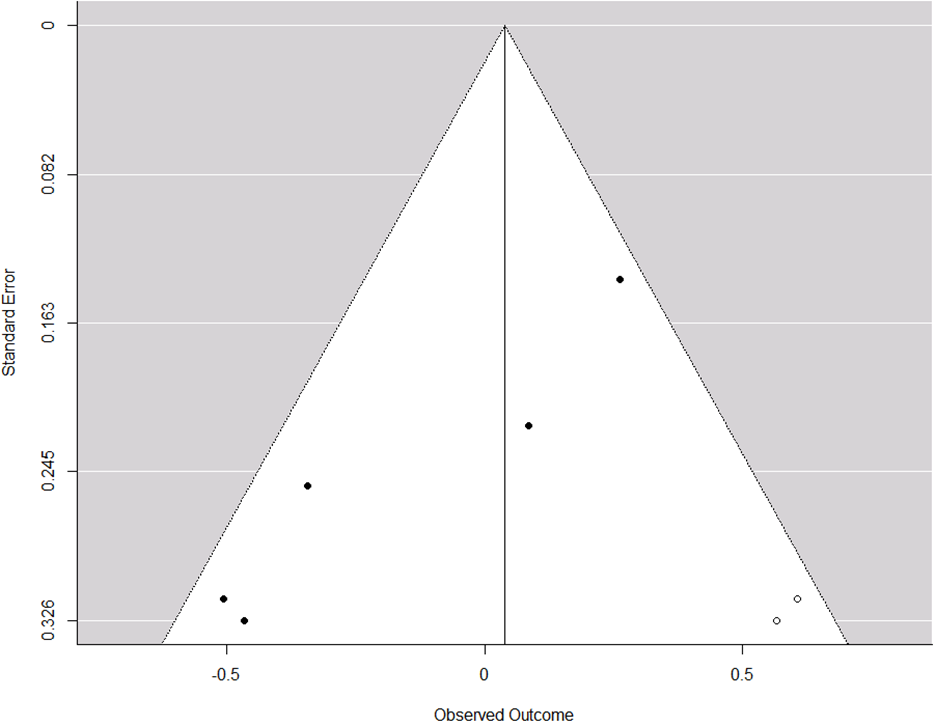


ESM3.7 High Support vs Floating Outreach Services – Wellbeing


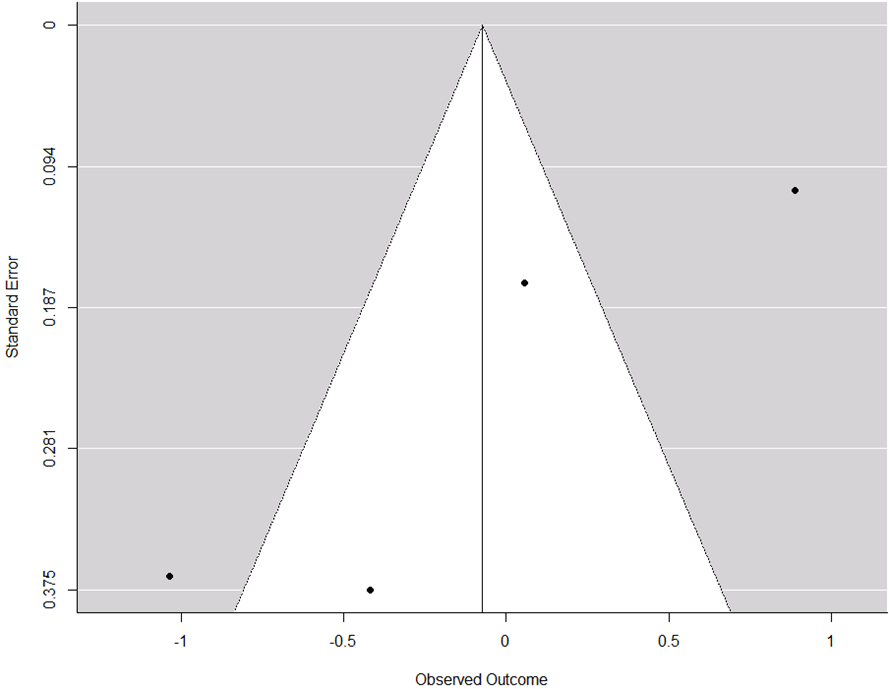


ESM3.8 High Support vs Floating Outreach Services – Living Conditions


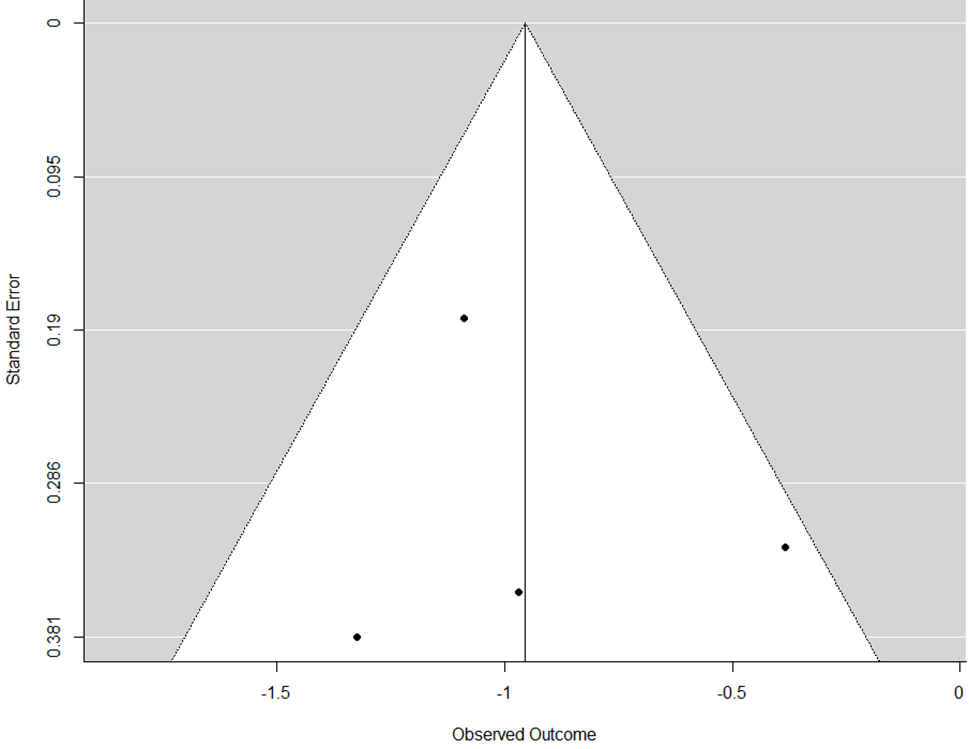


ESM3.9 High Support vs Floating Outreach Services – Social Functioning


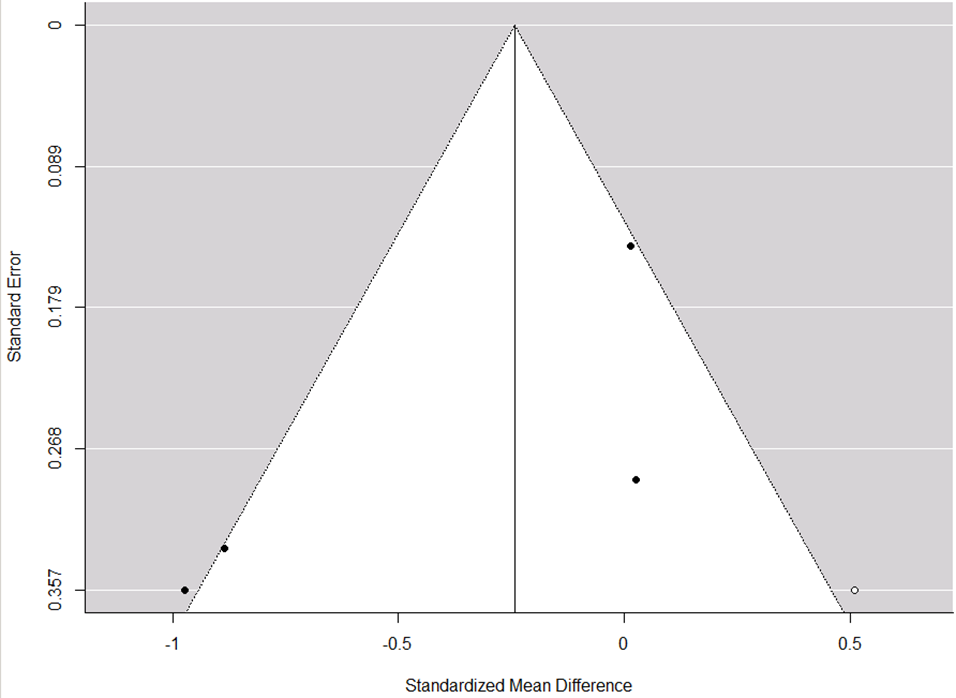


ESM4: Stratified Forest Plots: High Support vs Supported Housing


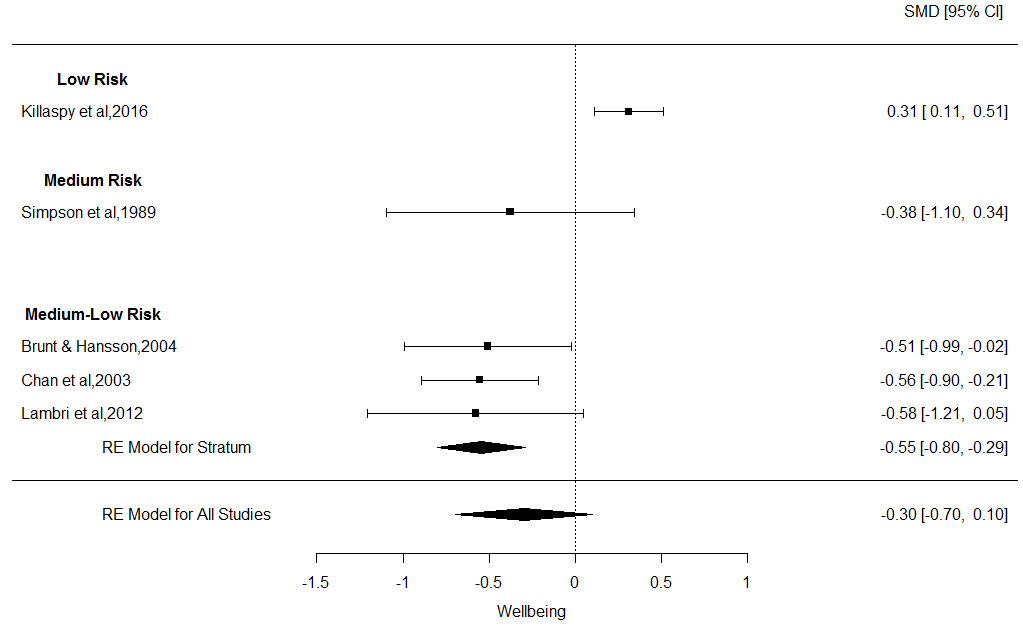


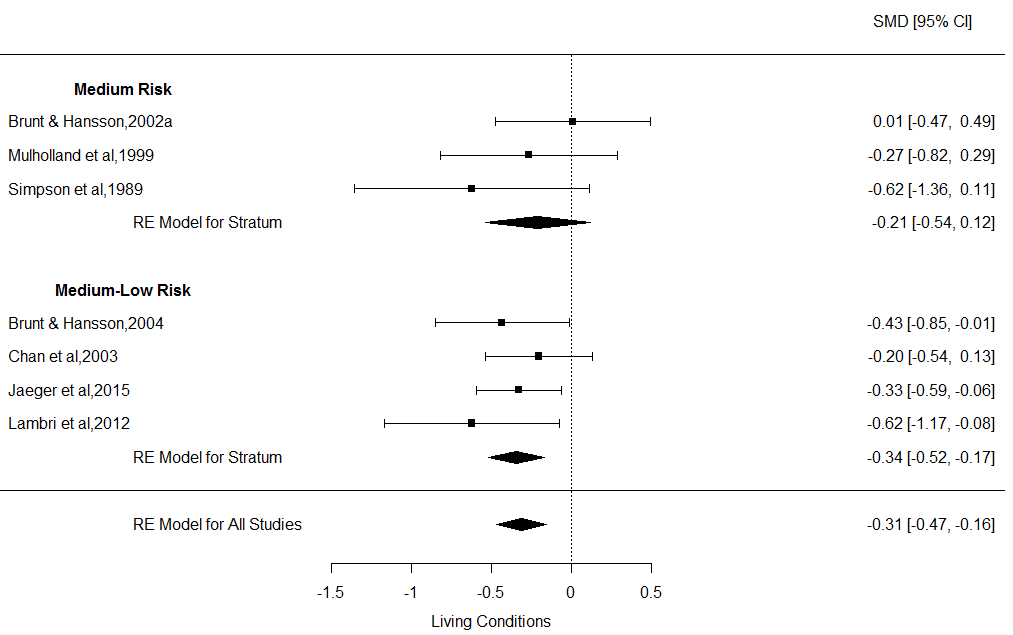


*
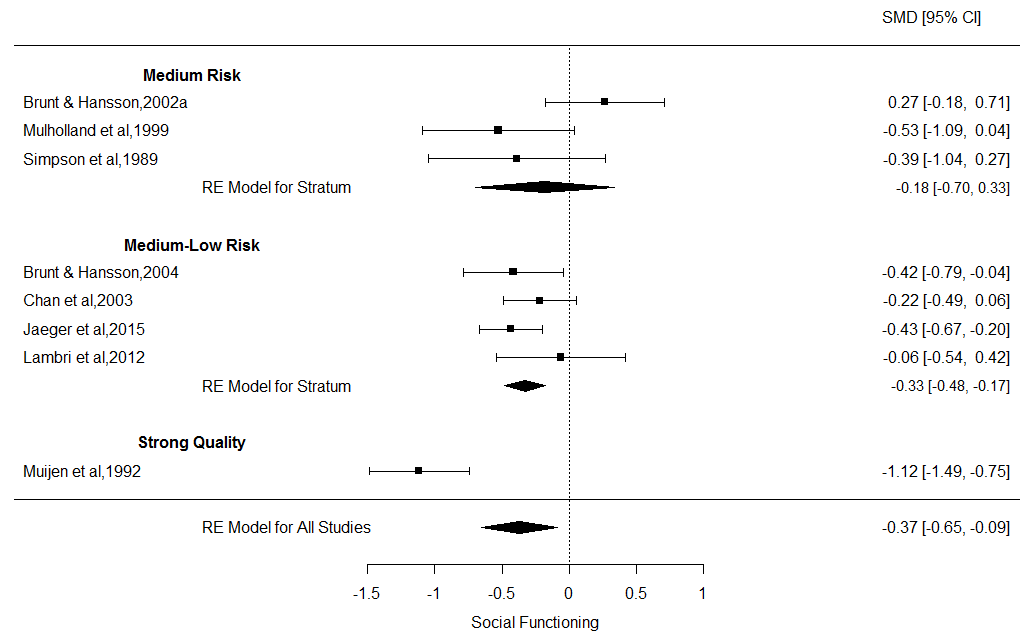
*

ESM5: Stratified Forest Plots: Supported Housing vs Floating Outreach

**
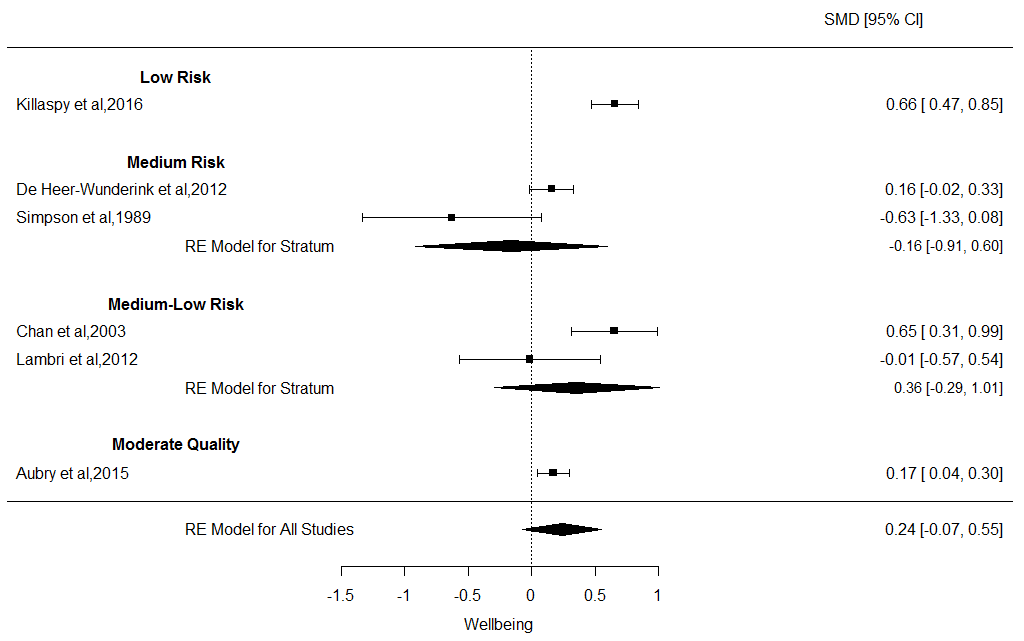
**


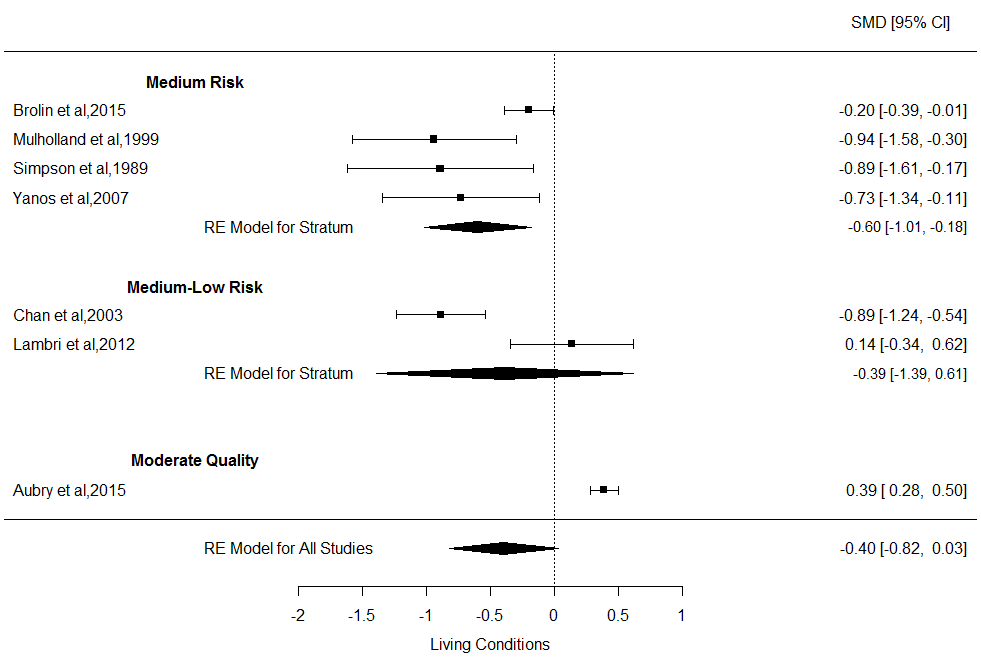


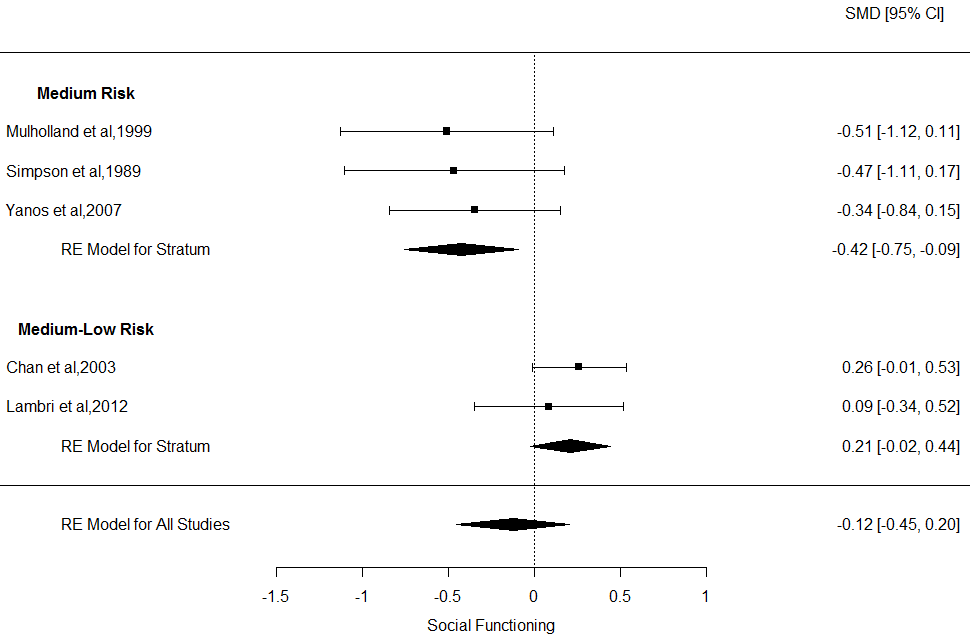


ESM6: Stratified Forest Plots: High Support vs Floating Outreach


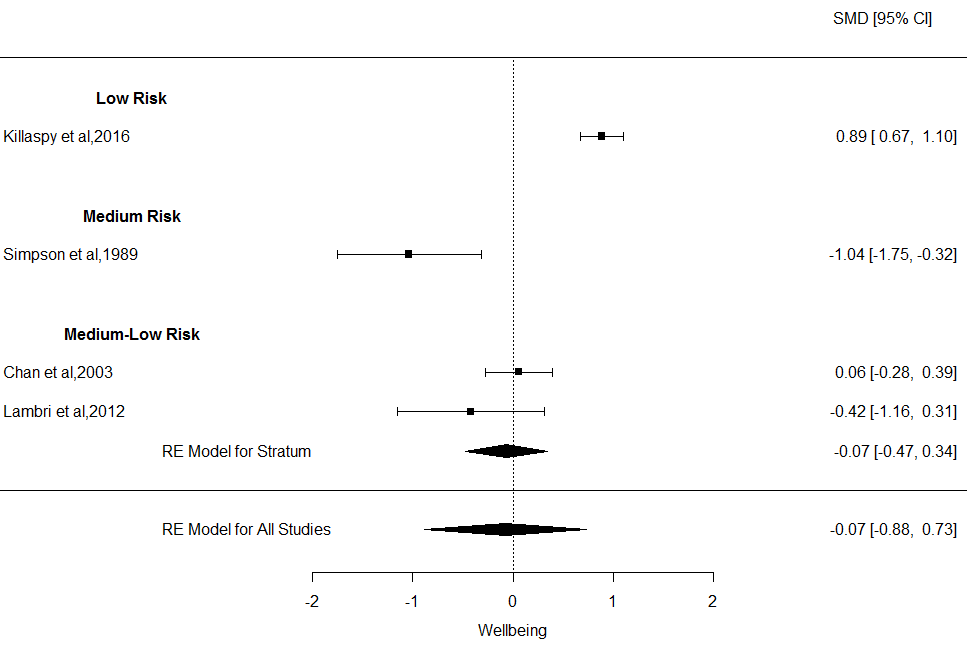


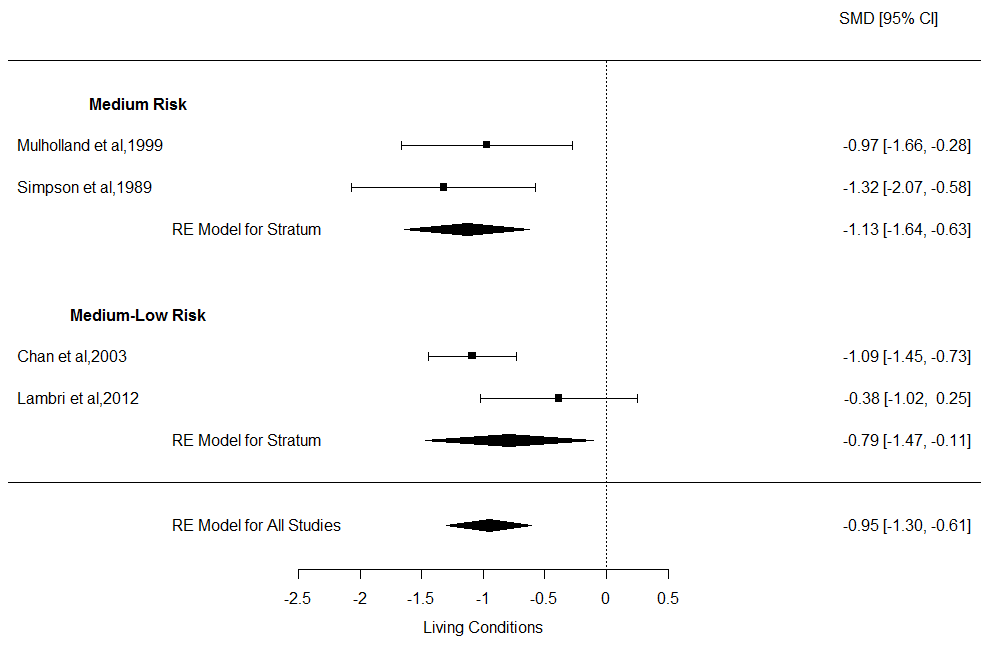


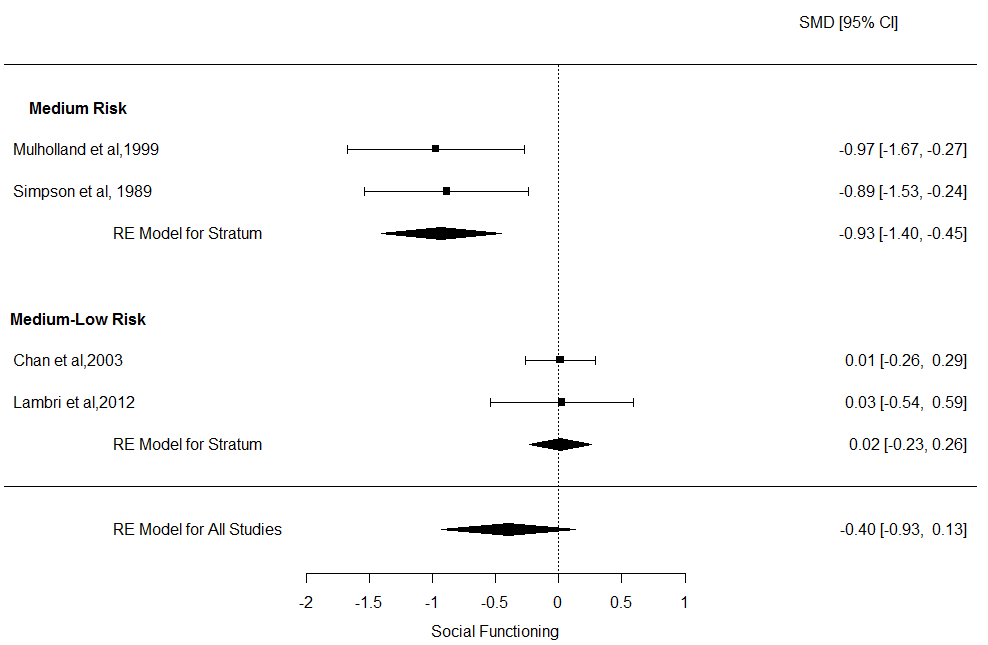


ESM7: Comparison of Living Conditions outcomes for individuals in High Support and Supported Housing


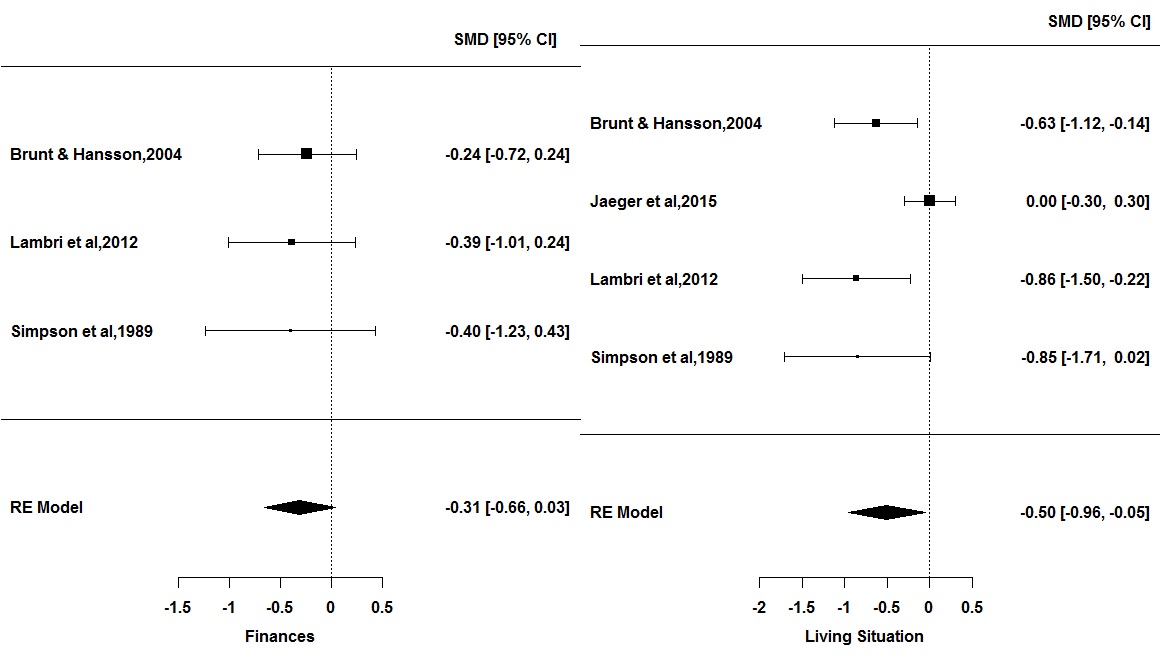


ESM8: Comparison of Social Functioning outcomes for individuals in High Support and Supported Housing


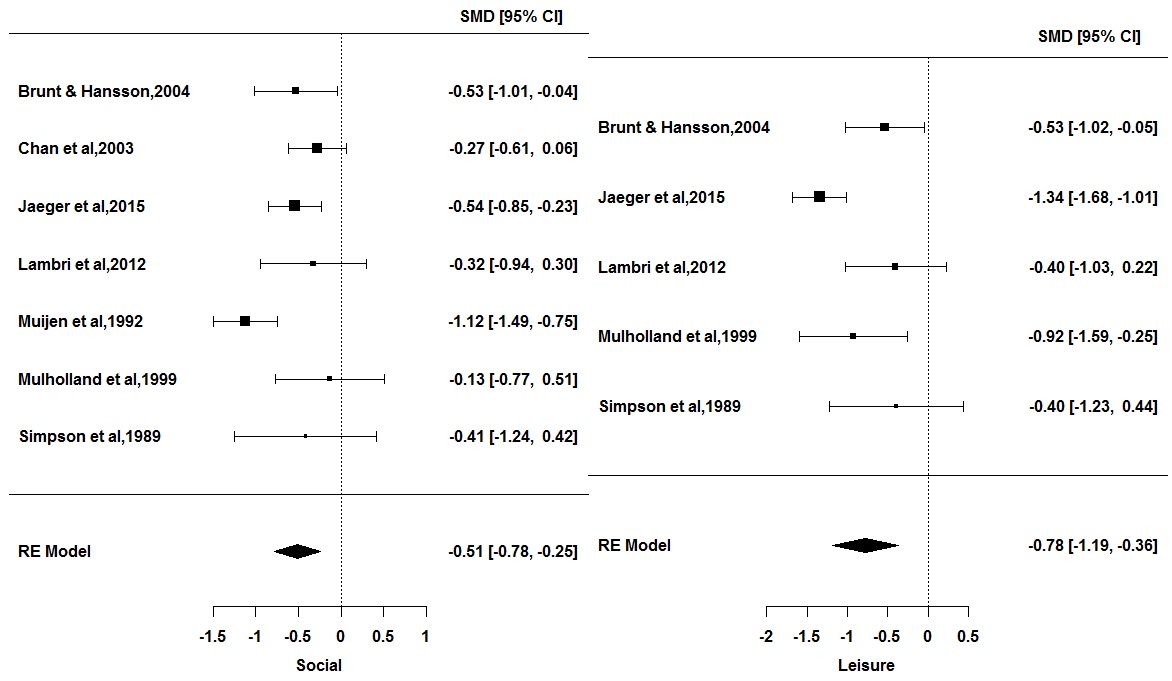


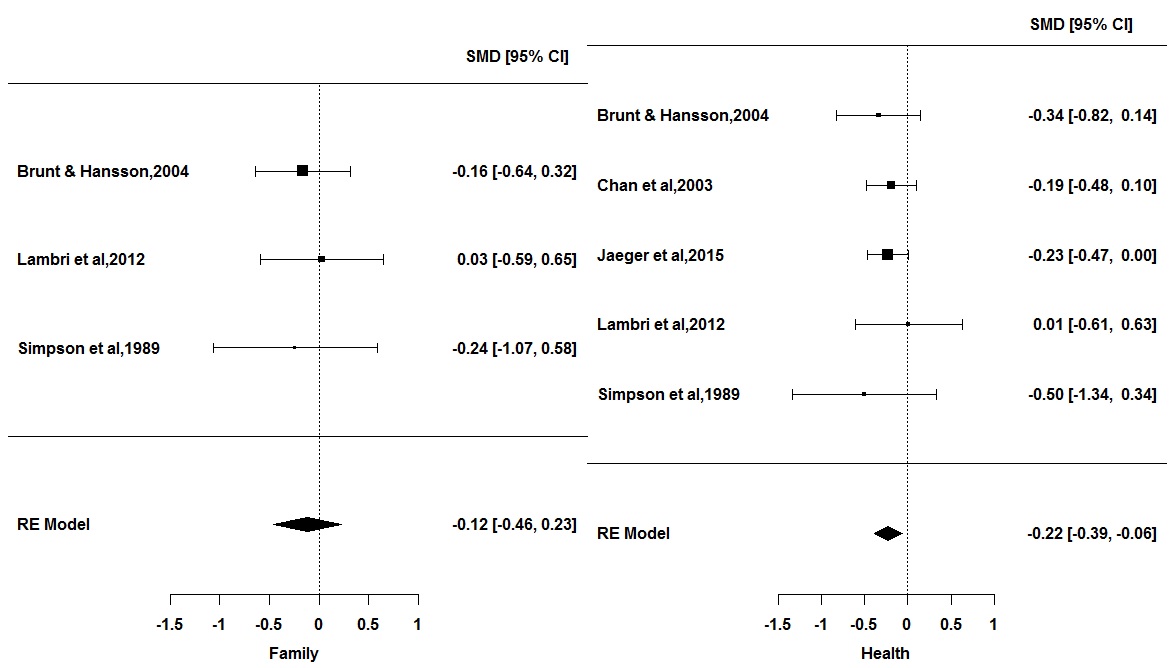


ESM9: Sensitivity analyses

ESM9.1 High Support vs Supported Housing – Wellbeing


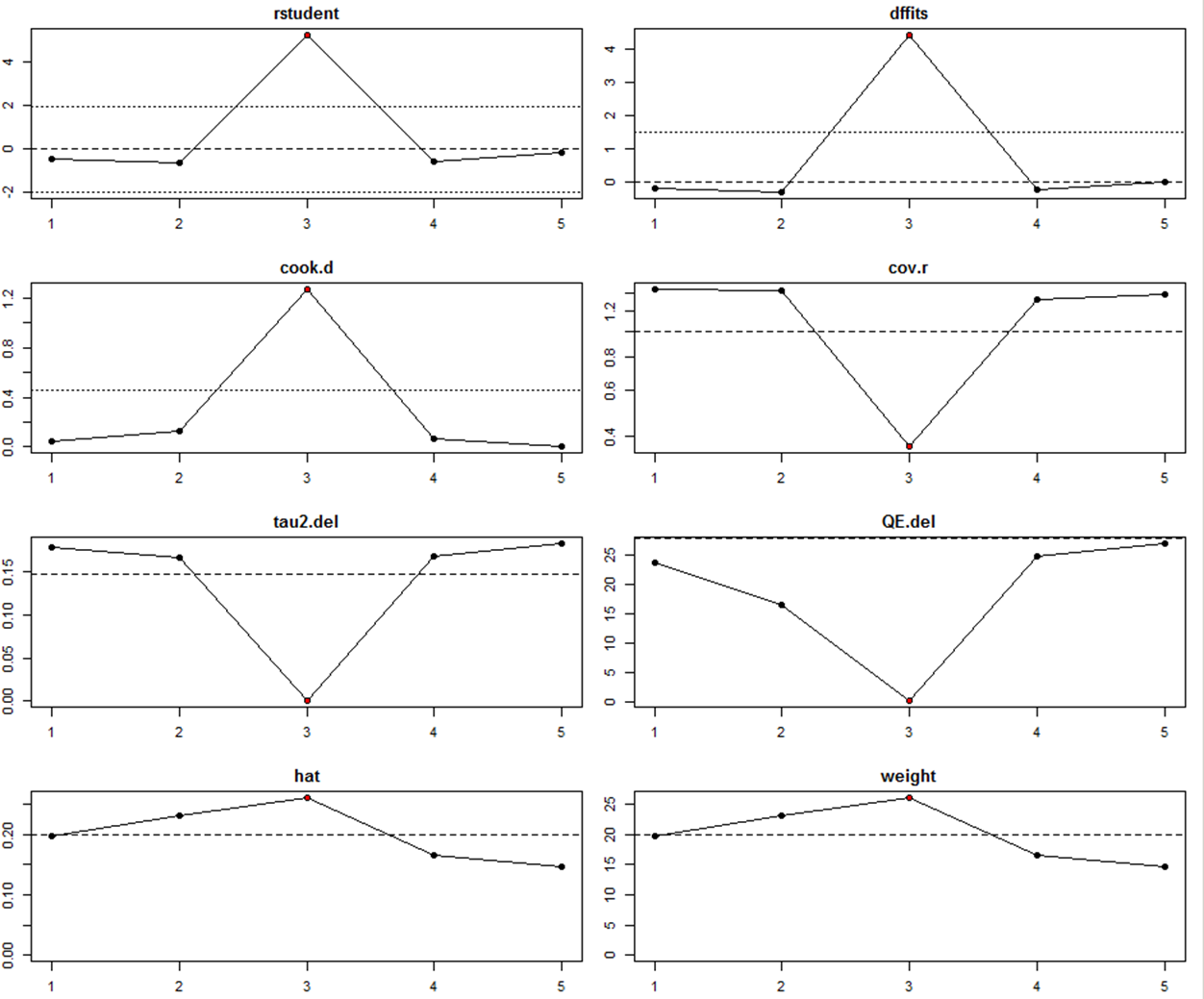


ESM9.2 High Support vs Supported Housing – Living Conditions


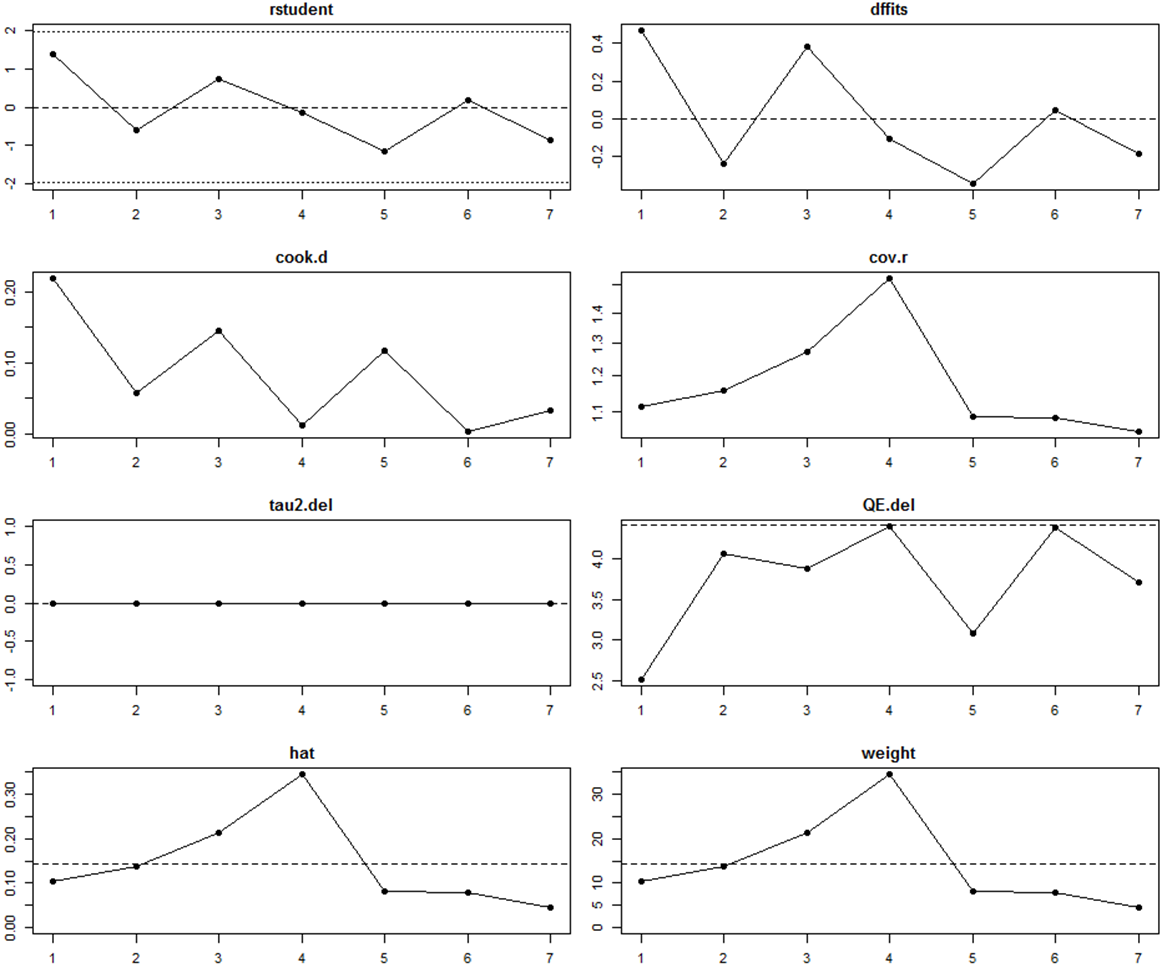


ESM9.3 High Support vs Supported Housing – Social Functioning


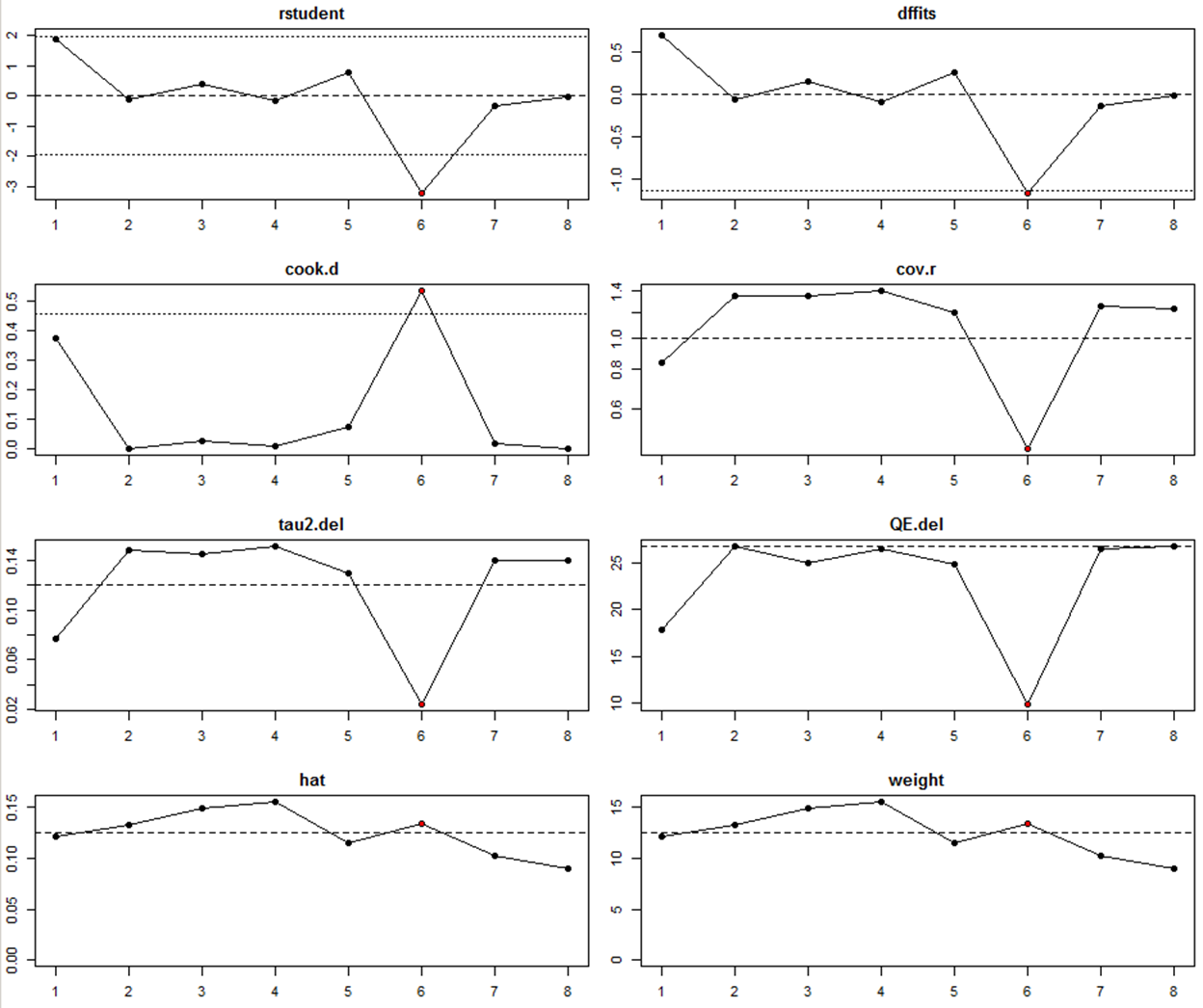


ESM9.4 Supported Housing vs Floating Outreach Services – Wellbeing


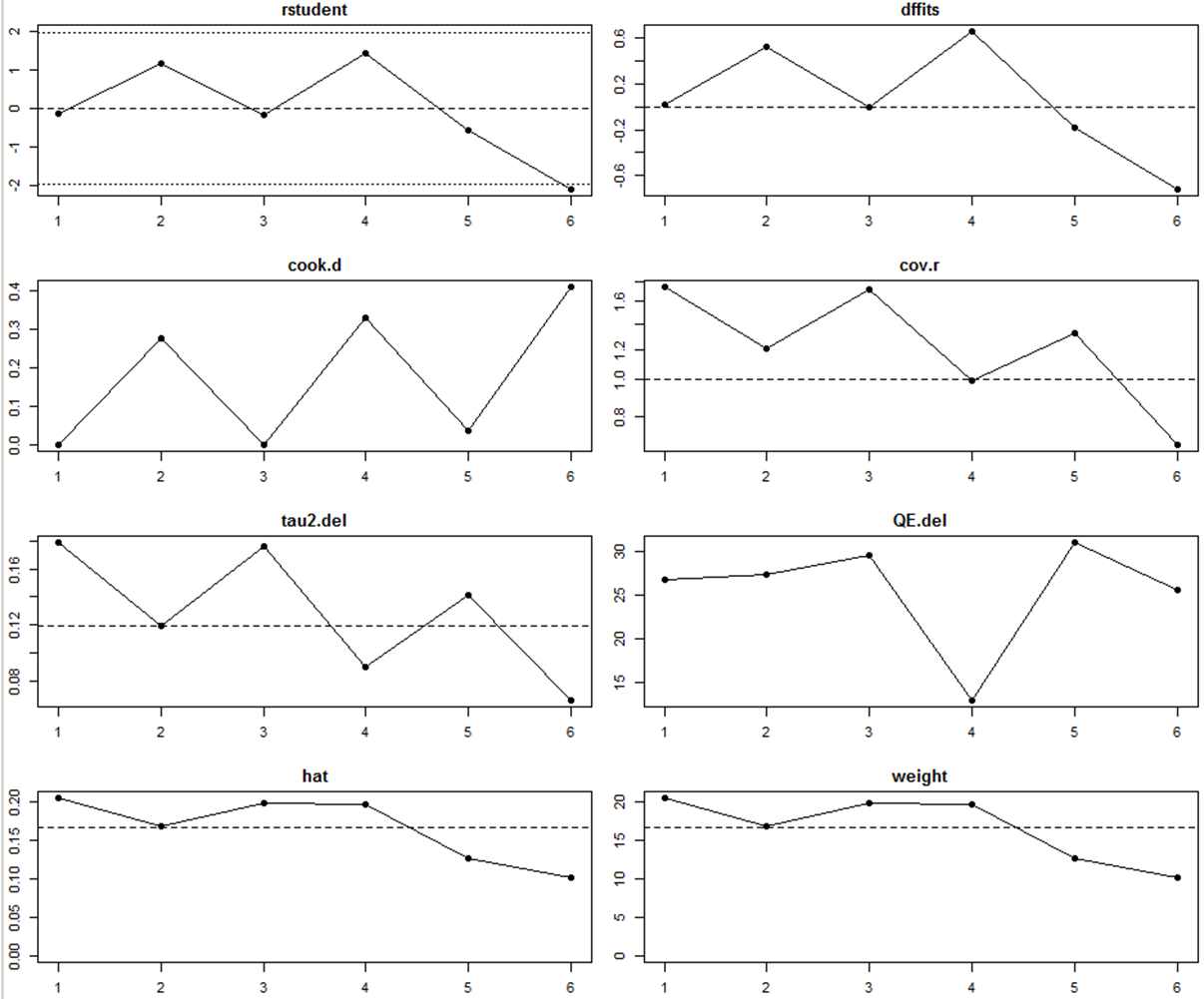


ESM9.5 Supported Housing vs Floating Outreach Services – Living Conditions


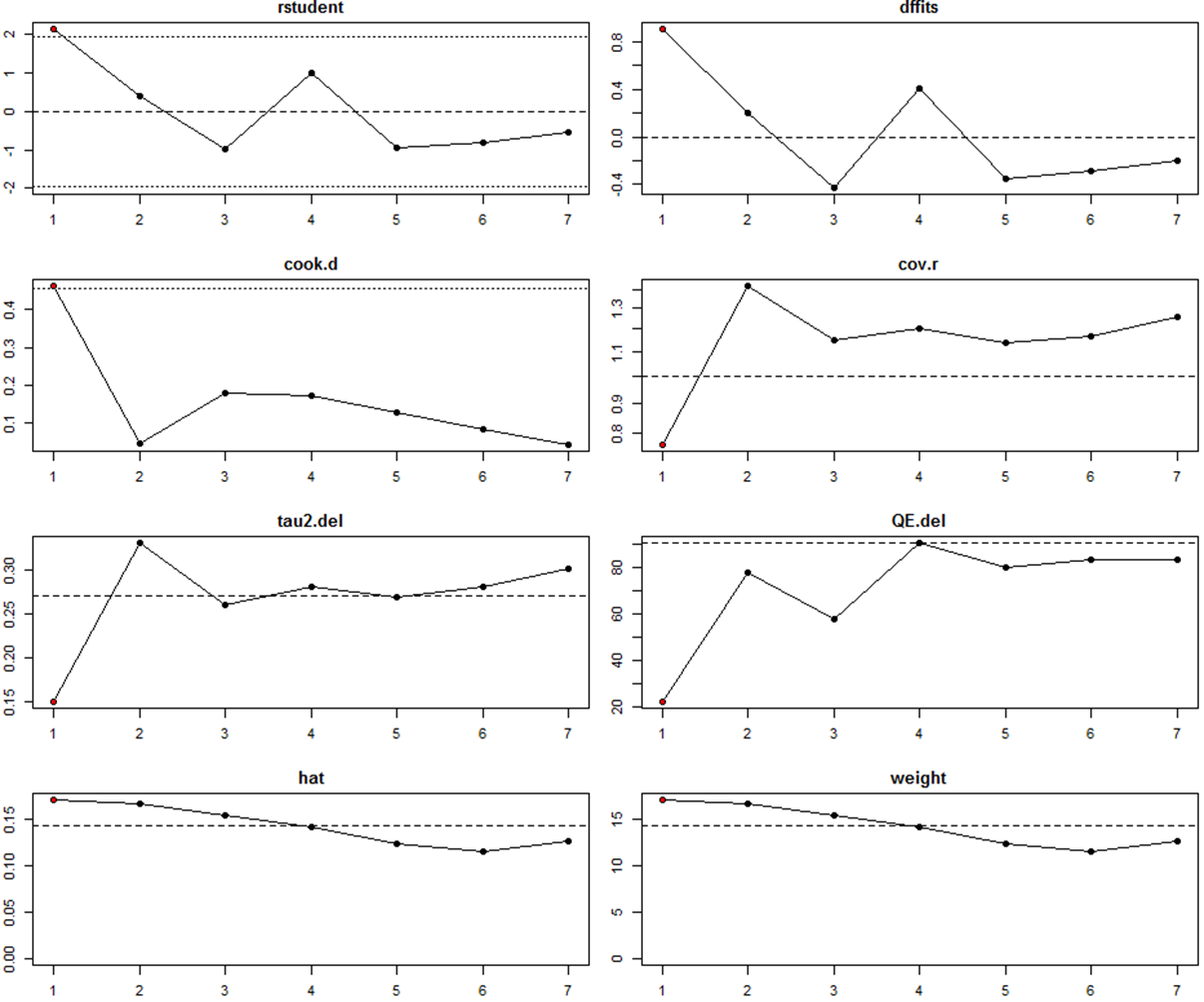


ESM9.6 Supported Housing vs Floating Outreach Services – Social Functioning


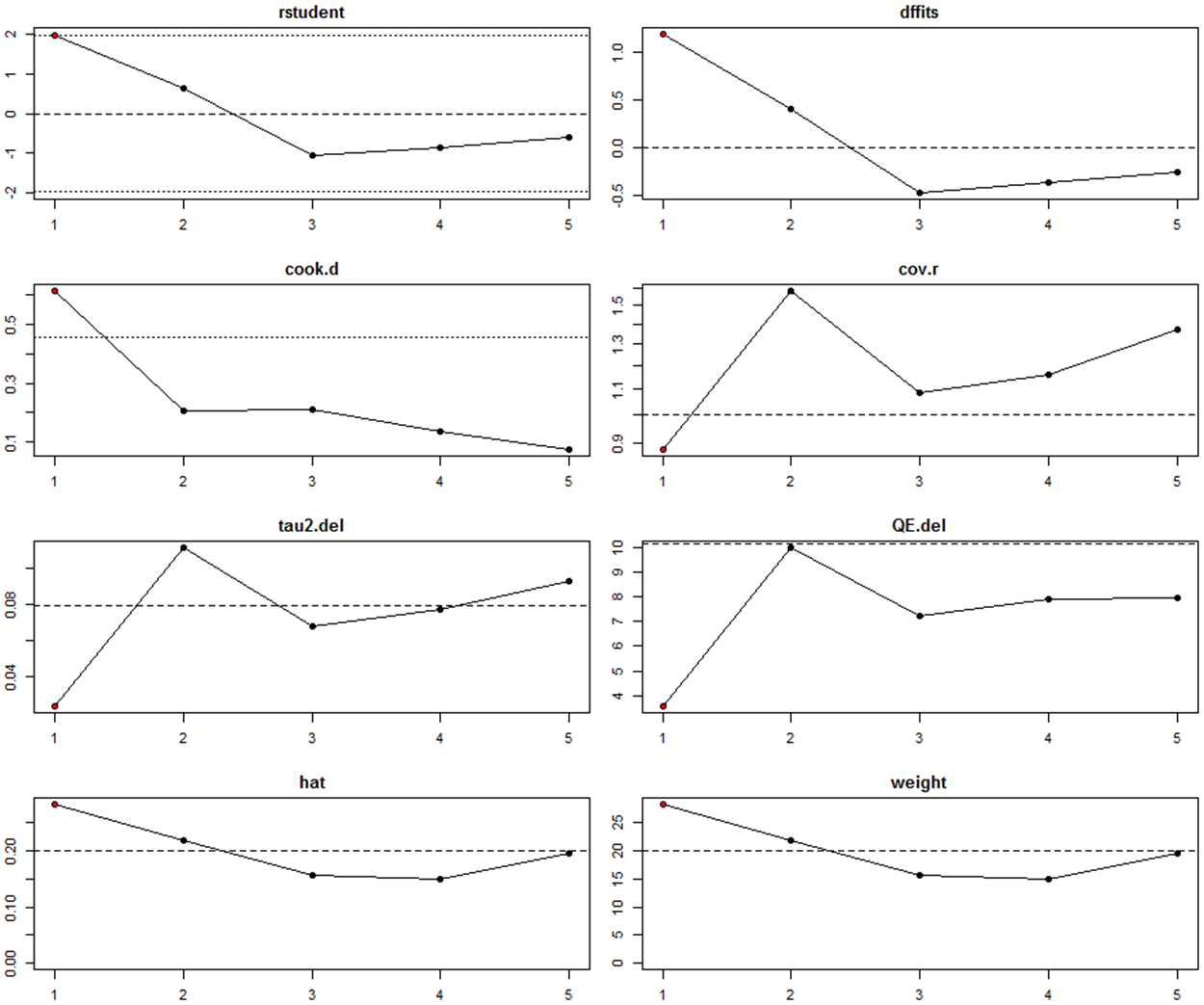


ESM9.7 High Support vs Floating Outreach Services – Wellbeing


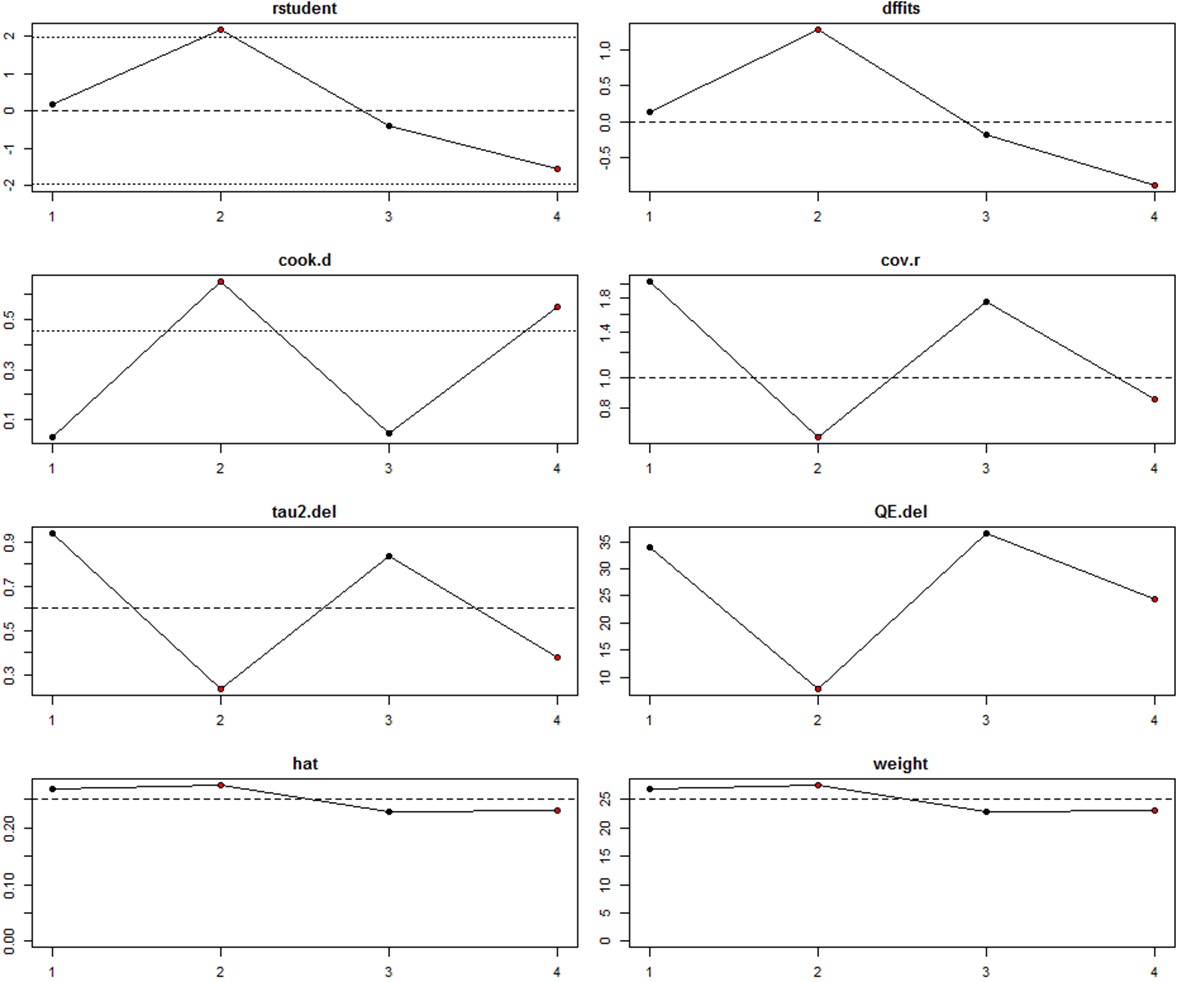


ESM9.8 High Support vs Floating Outreach Services – Living Conditions


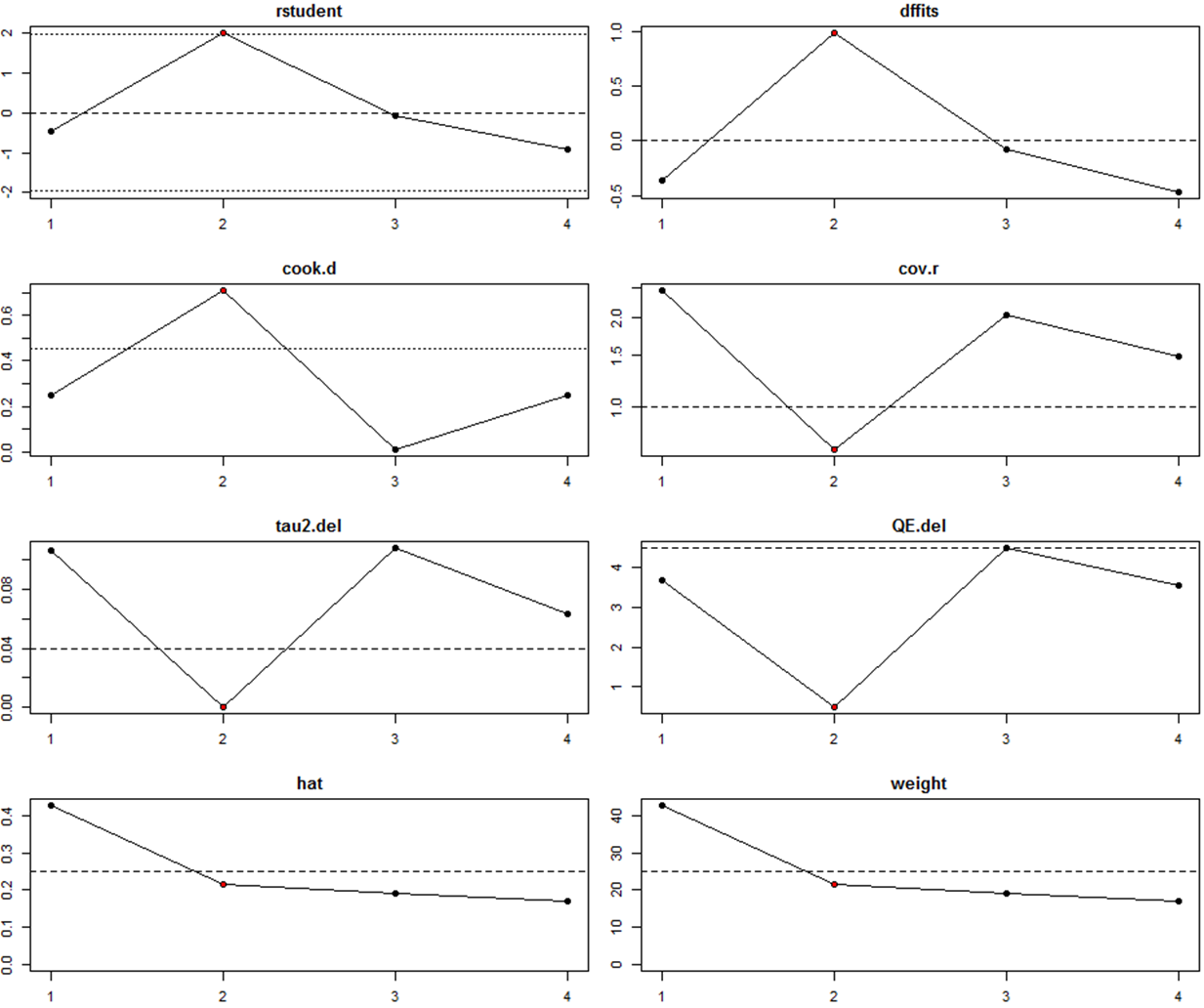


ESM9.9 High Support vs Floating Outreach Services – Social Functioning


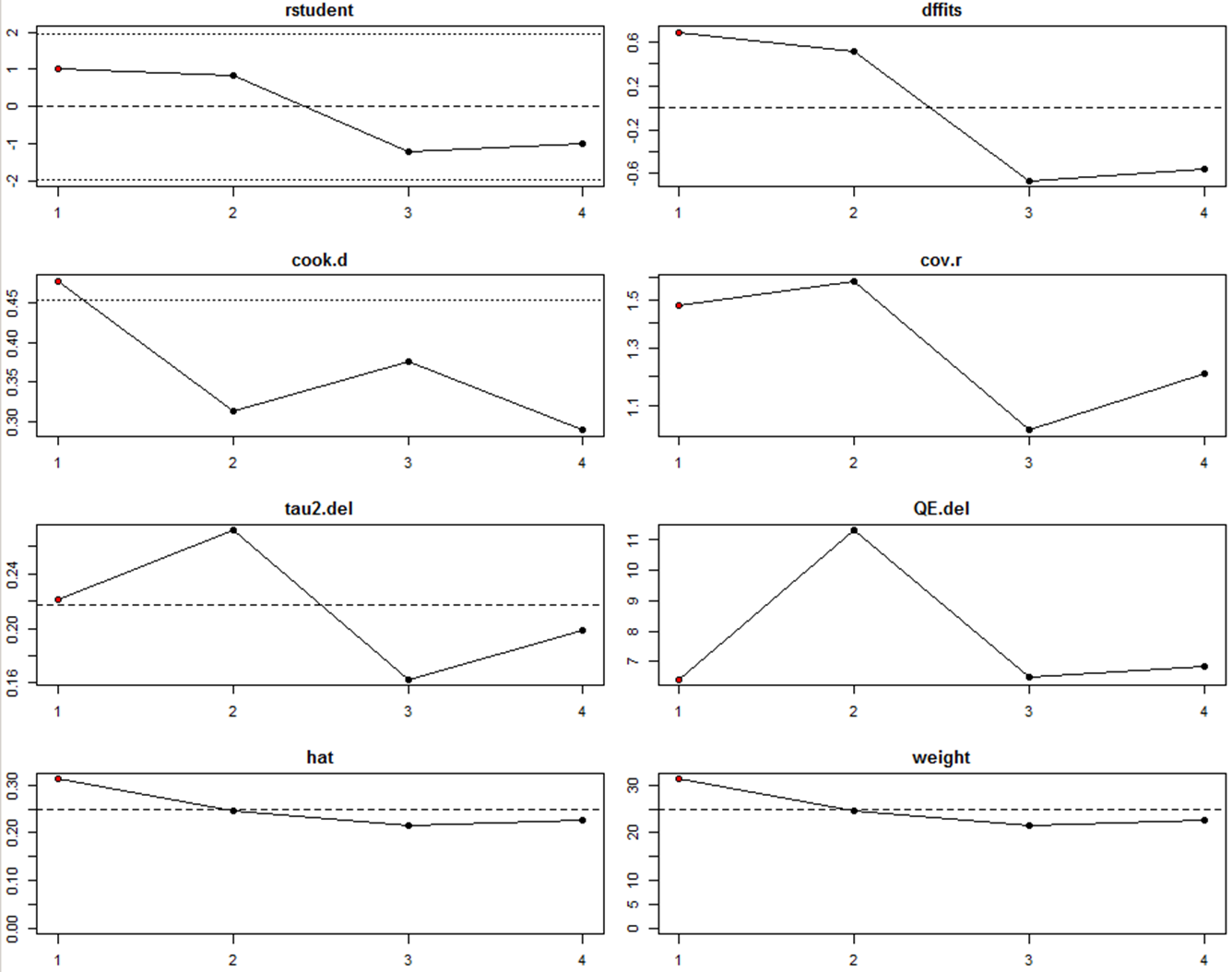

Supplement: Supplementary file 1 — Supplementary file1 (DOCX 2572 kb) [file 127_2020_1885_MOESM1_ESM.docx]
